# Evolution of digestive enzymes and dietary diversification in birds

**DOI:** 10.7717/peerj.6840

**Published:** 2019-04-25

**Authors:** Yan-Hong Chen, Huabin Zhao

**Affiliations:** Department of Ecology, Hubei Key Laboratory of Cell Homeostasis, College of Life Sciences, Wuhan University, Wuhan, China

**Keywords:** Birds, Diet, Digestive enzyme, Selection tests, Molecular evolution

## Abstract

As the most species-rich class of tetrapod vertebrates, Aves possesses diverse feeding habits, with multiple origins of insectivory, carnivory, frugivory, nectarivory, granivory and omnivory. Since digestive enzymes mediate and limit energy and nutrient uptake, we hypothesized that genes encoding digestive enzymes have undergone adaptive evolution in birds. To test this general hypothesis, we identified 16 digestive enzyme genes (including seven carbohydrase genes (hepatic* amy*, pancreatic* amy*, salivary* amy*, *agl*, *g6pc*, *gaa* and *gck*), three lipase genes (*cyp7a1, lipf* and *pnlip*), two protease genes (*ctrc* and *pgc*), two lysozyme genes (*lyz* and *lyg*) and two chitinase genes (*chia* and *chit1*)) from the available genomes of 48 bird species. Among these 16 genes, three (salivary* amy*, *lipf* and *chit1*) were not found in all 48 avian genomes, which was further supported by our synteny analysis. Of the remaining 13 genes, eight were single-copy and five (*chia*, *gaa*, *lyz*, *lyg* and *pgc*) were multi-copy. Moreover, the multi-copy genes *gaa*, *lyg* and *pgc* were predicted to exhibit functional divergence among copies. Positively selected sites were detected in all of the analyzed digestive enzyme genes, except *agl*, *g6pc*, *gaa* and *gck*, suggesting that different diets may have favored differences in catalytic capacities of these enzymes. Furthermore, the analysis also revealed that the pancreatic amylase gene and one of the lipase genes (*cyp7a1*) have higher *ω* (the ratio of nonsynonymous to the synonymous substitution rates) values in species consuming a larger amount of seeds and meat, respectively, indicating an intense selection. In addition, the *gck* carbohydrase gene in species consuming a smaller amount of seeds, fruits or nectar, and a lipase gene (*pnlip*) in species consuming less meat were found to be under relaxed selection. Thus, gene loss, gene duplication, functional divergence, positive selection and relaxed selection have collectively shaped the evolution of digestive enzymes in birds, and the evolutionary flexibility of these enzymes may have facilitated their dietary diversification.

## Introduction

Aves, as the largest class of tetrapod vertebrates, consists of approximately ten thousand known living species, of which more than half are passerines (Gill, 1995). Along with the rich diversity of species, birds have developed a diverse range of dietary habits. In terms of diet, birds can be roughly divided into seven categories: insectivores (referring to species that predominantly feed on insects, such as cuckoos and swifts), frugivores (species that mostly feed on fruits, such as mousebirds and manakins), granivores (birds that mostly feed on seeds of plants, such as finches and sandgrouses), carnivores (species that mostly eat non-insect animals, such as cormorants and eagles), folivores (birds that predominantly eat leaves, such as hoatzins), nectarivores (species that mostly feed on nectar, such as hummingbirds and sunbirds), and omnivores (species that feed on multiple food items, including insects, vertebrates, seeds, fruits, nectar or carrion, such as cranes and ravens) (Gill, 1995). Ancestral reconstruction indicated that seeds were one of the key food components in the most recent common ancestor of extant birds (Larson, Brown & Evans, 2016). Such diverse dietary habits have independently evolved multiple times in birds, and the enormous diversity in diet must demand different physiological adaptations to deal with various food items (Kissling, Sekercioglu & Jetz, 2012; Burin et al., 2016). Owing to such differences in avian food compositions, a number of digestive enzymes are required for nutrient degradation and digestion in birds (Martinez del Rio, Baker & Baker, 1992; Karasov, Martinez del Rio & Caviedes-Vidal, 2011).

There are several major categories of digestive enzymes (carbohydrases, proteases, lipases or esterases, and phosphatases or nucleases) that are expressed in digestive tracts and organs of vertebrates. Of the carbohydrases, α-amylases play essential roles in efficiently hydrolyzing longer chain polysaccharides (e.g., dietary starches) by acting on *α*-1,4-glycosidic bonds (Sogaard, Abe & Svensson, 1993), and it was indicated that passerines fed with higher starch diets had higher pancreatic amylase activities (Kohl et al., 2010). Furthermore, studies on phyllostomid bats have revealed that along with the shift from insectivory to nectarivory and frugivory, activities of the intestinal maltase and sucrase were significantly increased (Schondube, Herreram & Martinez del Rio, 2001). Notably, in nectarivorous, hover-feeding animals such as hummingbirds and bats, dietary sugar instead of fat is utilized as a premium fuel for efficiently supplying energy (Suarez & Welch, 2011).

Proteases are involved in digesting long-chain proteins into short fragments by attacking the peptide bonds linking two amino acid residues (Rawlings & Barrett, 1993). Signals of positive selection were identified at three protease genes (*ctrc*, *prss1* and *tmprss15*) separately encoding chymotrypsin C, serine protease 1 and transmembrane serine protease 15 in cetaceans, suggesting that cetaceans may have evolved an enhanced digestive capacity for proteins (Wang et al., 2016).

Lipases catalyze dietary triglycerides into monoglycerides and fatty acids (Houde, Kademi & Leblanc, 2004). Experiments on both the pine warbler (*Dendroica pinus*) and suckling goats indicated that individuals fed with diets containing the largest amounts of lipids showed the highest lipase activities (Lopez-Palomo et al., 1997; Levey et al., 1999).

Several digestive enzymes such as chitinases and lysozymes can hydrolyze chemical compounds such as chitin, one of the primary components of insect exoskeletons (Amano & Ito, 1978; Kramer & Muthukrishnan, 1997), and thus may be important in birds, since most birds eat insects and nearly all birds consume insects during their breeding seasons (Del Hoyo, Elliott & Sargatal, 1994). In addition, lysozymes can degrade chitin from fungi and peptidoglycan from bacteria (Berger & Weiser, 1957). Foregut fermenting species including langur, cow, and hoatzin are thought to utilize the c-type lysozyme to break down and assimilate nutrients from mutualistic microflora that synthesize cellulases to digest refractory plant materials (Stewart, Schilling & Wilson, 1987; Stewart & Wilson, 1987; Swanson, Irwin & Wilson, 1991; Kornegay, Schilling & Wilson, 1994). Functional assays in insectivorous bats suggested that of the two duplicates of lysozyme, one was expressed widely across different tissues, while the other was highly expressed in the tongue exclusively, and had a higher activity in degrading glycol chitin (Liu et al., 2014). In general, despite the increased attention that has been given to digestive enzymes in other vertebrates, molecular evolution of digestive enzymes across the avian phylogeny remains largely unknown.

In this study, we undertook evolutionary analyses of 16 digestive enzyme genes identified from 48 available avian genomes that represent nearly all avian orders (Hillier et al., 2004; Dalloul et al., 2010; Warren et al., 2010; Jarvis et al., 2014; Zhang et al., 2014), with an aim to reconstruct the evolutionary history of digestive enzyme genes across the avian phylogeny. Furthermore, selective pressure analyses were conducted to determine whether these digestive enzyme genes have undergone adaptive evolution associated with dietary diversification in birds, with an aim to test a series of evolutionary hypotheses (Table 1). We estimate *ω* (the ratio of nonsynonymous to synonymous substitution rates) as an indicator to examine changes in selective pressure among amino acid sites and along different lineages. A higher *ω* indicates a molecular signature of adaptive evolution. In the present study, we hypothesized that genes encoding amylases have a higher *ω* value in species consuming more grains, and that genes encoding carbohydrases (excluding amylases) have a higher *ω* value in species consuming more grains, fruits, or nectar. In a similar fashion, genes encoding lipases and proteases were hypothesized to have a higher *ω* value in species consuming more meat, and genes encoding lysozymes and chitinases were hypothesized to have a higher *ω* value in species consuming more insects (Table 1).

**Table 1:** Evolutionary hypotheses proposed in our molecular evolutionary analyses of the 16 avian digestive enzymes. Full names, Enzyme Commission (EC) numbers, sites of secretion, and digestive functions of these enzymes were also listed.

**Enzyme**	**Full name (EC number)**	**Site of secretion**^a^	**Digestive function**^a^	**Hypothesis**
**Carbohydrases**				
hepatic AMY	hepatic amylase (3.2.1.1)	liver	Facilitates the hydrolysis of starch	Species eating more grains have a higher *ω* value
pancreatic AMY	pancreatic amylase (3.2.1.1)	pancreas	Facilitates the hydrolysis of starch	
salivary AMY	salivary amylase (3.2.1.1)	salivary gland	Facilitates the hydrolysis of starch	
AGL	glycogen debranching enzyme (2.4.1.25)	muscle, liver, heart	Participates in the breakdown of glycogen	Species eating more grains, fruits, or nectar have a higher *ω* value
G6PC	glucose-6-phosphatase, catalytic subunit (3.1.3.9)	liver, kidney and intestine	Facilitates the hydrolysis of glucose-6-phosphate in gluconeogenesis	
GAA	α-1,4-glucosidase (3.2.1.20)	intestine	Participates in glycogen hydrolysis	
GCK	glucokinase (2.7.1.1)	gastrointestinal tract, liver, pancreas	Facilitates phosphorylation of glucose to glucose-6-phosphate	
**LIPASES/ESTERASES**				
LIPF	gastric lipase (3.1.1.3)	stomach	Initiates the digestion of triglycerides	Species eating more meat have a higher *ω* value
CYP7A1	cholesterol 7-α-hydroxylase (1.14.14.23)	liver	Participates in bile acid synthesis	
PNLIP	pancreatic lipase (3.1.1.3)	pancreas	Hydrolyses ester linkages of triglycerides	
**PROTEASES**				
CTRC	chymotrypsin C (3.4.21.2)	pancreas	Hydrolyses peptide bonds involving phenylalanine, tyrosine, and tryptophan	Species eating more meat have a higher *ω* value
PGC	progastricsin (3.4.23.3)	stomach	Hydrolyses peptide bonds involving phenylalanine, tyrosine, and leucine	
**LYSOZYMES**				
LYZ	c-type lysozyme (3.2.1.17)	gastrointestinal tract, eggs, blood	Cleaves the β-1,4-glycosidic bond between NAM and NAG in peptidoglycan	Species eating more insects have a higher *ω* value
LYG	g-type lysozyme (3.2.1.17)	intestine, tongue, eggs	Cleaves the β-1,4-glycosidic bond between NAM and NAG in peptidoglycan	
**CHITINASES**				
CHIT1	chitinase 1 (3.2.1.14)	bone marrow, and lung	Participates in degrading chitin	Species eating more insects have a higher *ω* value
CHIA	acidic mammalian chitinase (3.2.1.14)	gastrointestinal tract, tongue and kidney	Participates in degrading chitin	

**Notes.**

a Descriptions on sites of secretion and digestive functions were derived from Bao et al. (1997), Benkel et al. (2005), Boot et al. (1995); Boot et al. (2001), Carriere et al. (1993), Desnuelle (1960), Gratecos & Desnuelle (1971), Hornbuckle, Simpson & Tennant (2008), Irwin & Gong (2003), Jetton et al. (1994), Lindsay (1984), Matschinsky & Ellerman (1968), McKenzie (1996), Myant & Mitropoulos (1977), Nordlie & Sukalski (1985), Pan et al. (1998), Swaminathan & Radhakrishnan (1965), Taggart et al. (1989), Walker & Rao (1964) and Whitcomb & Lowe (2007).

## Materials & Methods

### Dietary data

**Figure 1:**
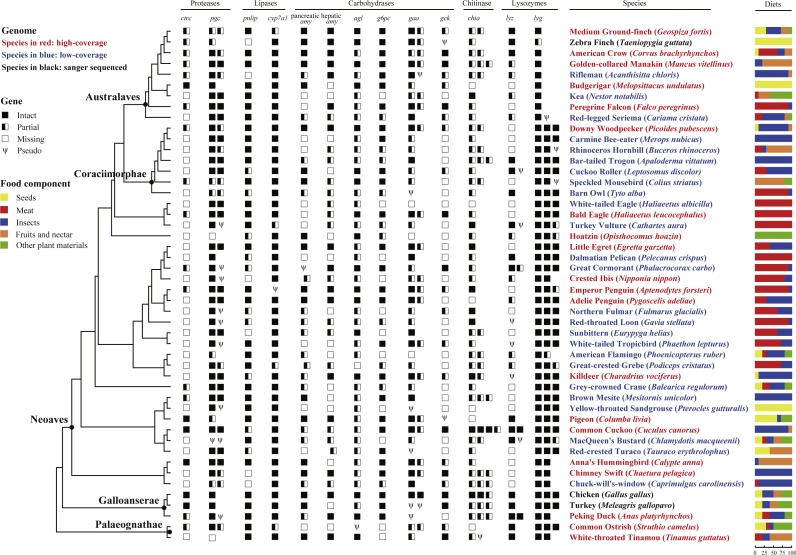
Survey of 13 digestive enzyme genes in 48 birds. The phylogeny of 48 birds adapted from previous phylogenetic analysis (Prum et al., 2015), is shown on the left with species names indicating genome information: high-coverage (red), low-coverage (blue) or Sanger-sequenced (black); higher taxon names mentioned in the text are shown at selected nodes; branch lengths were not proportional to the evolutionary time. Digestive enzyme genes identified from 48 avian genomic sequences are shown in symbols, where solid boxes represent intact genes, semi-open boxes represent partial genes, open boxes indicate undetected genes, and the Greek letter psi (Ψ) indicate pseudogenes. For each species, percentages of food composition are displayed on the right, with yellow, red, blue, orange and green separately representing seeds, meat, insects, fruits and nectar, and other plant materials in diets.

Dietary information of 48 bird species (Fig. 1 and Table S1) was derived from a comprehensive diet database. This database transformed the verbal dietary description derived from many key literature sources, such as handbooks and monographs, into standardized and semiquantitative information about relative importance of different food categories (Wilman et al., 2014). Five food categories consumed by birds were classified (including insects, meat, seeds, fruits or nectar, and other plant materials) (Fig. 1 and Table S1). The mean of consumption for a particular food component in all 48 birds with available genome sequences (Jarvis et al., 2014; Zhang et al., 2014) was calculated as 12.71% (seeds), 31.04% (meat), 34.38% (insects), and 25.42% (seeds, fruits and nectar), respectively. We assigned a species to a high or low group depending on whether its consumption is higher or lower than the mean. For details, see Supplemental Material S1.

### Selection of digestive enzyme genes

Numerous digestive enzyme genes are involved in various catabolic or hydrolytic pathways and play important roles in organic substance degradation and nutritional uptake. For proteases, PGC and CTRC independently secreted by the gastric chief cell and the pancreas, have been demonstrated for their critical functions in gastrointestinal digestion of proteins (Wilcox, 1970; Dunn, 2001). Lipases in the stomach (LIPF) and pancreas (PNLIP), and the rate-limiting enzyme in bile acid syntheses, CYP7A1, have been shown to play important roles in digestion and absorption of lipids (Vallim, Tarling & Edwards, 2013; Sitrin, 2014). Because dietary components of many bird species contain certain amounts of seeds, fruits, nectar and insects, several carbohydrases (e.g., amylase, hexokinase and glucosidase) and enzymes involved in digesting insects (e.g., chitinase and lysozyme) were selected.

Ultimately, a total of 16 digestive enzyme genes [seven carbohydrase genes (salivary *amy*, hepatic *amy*, pancreatic *amy*, *agl*, *g6pc*, *gaa* and *gck*), three lipase genes (*cyp7a1*, *lipf* and *pnlip*), two protease genes (*ctrc* and *pgc*), two lysozyme genes (*lyz* and *lyg*) and two chitinase genes (*chia* and *chit1*)] were selected as key genes in digesting sugars, lipids, protein and insects (Karasov, Martinez del Rio & Caviedes-Vidal, 2011). Our selection of enzymes included two criteria: molecular structures and functions of the enzymes are well studied, and representatives of major categories of digestive enzymes are included. The Enzyme Commission (EC) numbers, sites of secretion and digestive functions of all selected enzymes are listed in Table 1.

### Identification of digestive enzyme genes

From the same 48 avian species whose dietary data were collected, genomes sequences were retrieved from the Avian Phylogenomics Project (http://avian.genomics.cn/en/) (Jarvis et al., 2014; Zhang et al., 2014), and the basic statistics for each assembly are listed in Table S2. To identify the 16 digestive enzyme genes from each genome, we followed a method used in an earlier study (Wang & Zhao, 2015) with minor modifications. Synteny analysis was undertaken to further determine whether those undetected digestive enzyme genes are truly lost by querying their adjacent genes, with human and chicken genomes as references. The deduced nucleotide sequences of digestive enzyme genes, which contain all pseudogenes as well as partial and intact genes, are provided in Dataset S1. See also Supplemental Material S1.

### Phylogenetic analysis

Phylogenetic trees based on each multi-copy gene were reconstructed with the Bayesian approach using the program MrBayes version 3.1.2 (Ronquist et al., 2012) with five million generations. The best-fitting models of nucleotide substitution were selected with the smallest Akaike Information Criterion (AIC) (Akaike, 1974) for each multi-copy gene, using the program MrModelTest version 2.3 (Nylander, 2004). See also Supplemental Material S1.

### Evolutionary changes of gene family sizes

In order to understand evolutionary changes in the number of multi-copy digestive enzyme genes across the avian phylogeny, we used the program Computational Analysis of gene Family Evolution (CAFE) version 4.0 (De Bie et al., 2006), which also estimates the error rate associated with the gene copy number in a gene family and evaluates the quality of the genomic information (e.g., assembly and annotation) (Hahn et al., 2005).

Using the phylogenetic tree derived from a recent study (Prum et al., 2015) and the data of gene family sizes in the five multi-copy digestive enzyme genes (*gaa*, *pgc*, *chia*, *lyz*, and *lyg*) from the 48 extant bird species with a sequenced genome, we inferred the global birth and death rates of the five gene families and the gene family sizes at each ancestral node, and identified rapidly evolved branches with accelerated rates of gene gain and loss (*P* value <  0.01) (De Bie et al., 2006).

### Selection pressure analyses

To test whether differences in dietary habits have driven adaptive evolution of digestive enzyme genes in birds, we undertook a series of analyses to detect positive selection and differences in selection intensity.

First, two site-specific models in the Phylogenetic Analysis by Maximum Likelihood (PAML) package (Yang, 2007), M8a and M8 (Swanson, Nielsen & Yang, 2003), were used to detect positively selected sites in each gene, whereby *ω* (the ratio of nonsynonymous to synonymous substitution rates) can vary among amino acid sites, with *ω* < 1, *ω* = 1 and *ω* > 1 indicating negative selection, neutral evolution and positive selection, respectively. M8 is the alternative model, which assumes a beta distribution for *ω* among sites (0 < *ω* < 1) and an extra class of sites with positive selection (*ω*>1), while M8a is the null model, which assumes a beta distribution for *ω* among sites (0 < *ω* < 1) and an extra class of sites with neutral evolution (*ω* = 1). Furthermore, three improved likelihood methods in the Datamonkey web server, with both nonsynonymous and synonymous substitution rates under consideration (Pond & Frost, 2005), were used to further evaluate the signals of positive selection. Sites that were predicted to be under positive selection in this study were detected by at least two of the four likelihood methods. See also Supplemental Material S1.

Second, the branch model performed through CODEML in the PAML package (Yang, 2007) was used to estimate *ω* values among branches. Different *ω* values were compared between the two groups of branches, one containing species with a higher consumption of particular food items and the other containing species with a lower consumption of the same particular food items (Table S3). See also Supplemental Material S1.

In addition to several approaches described above for detecting branches or sites under positive selection, the RELAX method (Wertheim et al., 2015) in the HyPhy package (Pond, Frost & Muse, 2005) was used to test whether natural selection was intensified or relaxed along any of the branches in the avian phylogeny. Here, we hypothesized that selection intensity was relaxed on lineages with (1) a lower meat consumption for proteases and lipases, (2) a lower seed consumption for amylases, (3) a lower consumption of seeds, fruits or nectar for carbohydrases (excluding amylases), and (4) a lower insect consumption for chitinases and lysozymes. See also Supplemental Materials S1.

### Functional divergence testing

Among the five multi-copy genes (*chia*, *gaa*, *lyc*, *lyg* and *pgc*), the gene tree reconstructed with *chia* sequences was unable to form distinct sub-clusters (Fig. S1), thus functional divergence between sub-clusters of each of the four multi-copy genes (*gaa*, *lyz*, *lyg* and *pgc*) was inferred by the program DIVERGE version 3 (Gu & Vander Velden, 2002). Functional divergence can be divided into two types: type-I and type-II. Type-I suggests altered functional constraints (such as different evolutionary rates), whereas type-II indicates radical changes in amino acid biochemical properties (such as charge positive/negative, hydrophilic/hydrophobic) between two gene clusters. See also Supplemental Material S1.

### Ancestral state reconstruction

We reconstructed the ancestral characters in Mesquite version 3.6 (Maddison & Maddison, 2018) using the parsimony model to determine the dietary composition of ancestral lineages and infer the evolutionary history of dietary diversification across the avian phylogeny. The proportions of a specified dietary component such as seeds, meat, fruits and nectar, or insects, were regarded as continuous characters. The phylogenetic tree representing the branching history of descent avian taxa was derived from Prum et al. (2015).

## Results

### Identification of digestive enzyme genes

Using published vertebrate protein sequences of each digestive enzyme gene as queries, we executed tblastn searches and identified target genes from 48 avian genome sequences (Fig. 1), which cover all but three orders of the Aves (Jarvis et al., 2014; Zhang et al., 2014). For convenience, all identified sequences of each gene were classified into three categories: intact genes, partial genes and pseudogenes, among which the first two categories were putatively functional and pseudogenes resulting from nonsense or frame-shifting mutations were regarded as possibly nonfunctional.

Of the 16 digestive enzyme genes, three (*chit1*, *lipf* and salivary *amy*) were not found in 48 avian genome sequences. Synteny analysis on each of these three genes showed that although both upstream and downstream genes were found to be located on the same scaffold or chromosome in most species, the corresponding digestive enzyme gene could not be detected, suggesting an absence in the common ancestor of birds regardless of their dietary habits (Table S4). The remaining 13 genes are all composed of multiple exons with exon number ranging from 4 in *lyz* to 33 in *agl*, and lengths of coding regions range from 444 bp in *lyz* to 4,593 bp in *agl*. Among these, eight (*agl*, *ctrc*, *cyp7a1*, *g6pc*, *gck*, hepatic *amy*, pancreatic *amy* and *pnlip*) are single-copy genes, while the remaining five (*chia*, *gaa*, *lyg*, *lyz* and *pgc*) are multi-copy genes, with copy numbers ranging from two to four (Fig. 1). Notably, due to incomplete genome sequencing or possible gene loss, we were able to identify only 6 and 12 intact sequences in the *ctrc* and *chia* genes, respectively, with the former being absent in 27 avian genome sequences and the latter being incomplete in 37 avian genome sequences (Fig. 1).

### Loss and duplication of digestive enzyme genes

Each digestive enzyme gene identified from avian genome sequences showed a high level of sequence similarity. All identified sequences of one digestive enzyme gene possessed an identical number of exons, except the triplicated *lyg* gene, wherein one copy *lygC* appears to have lost the first exon compared to the other two copies (*lygA* and *lygB*) (Fig. 1); this observation was first identified through immunoblots by Nile et al. (2004). These findings suggested that all digestive enzyme genes are evolutionarily conserved and thus functionally important in birds.

Phylogenetic analyses were undertaken to examine events of gene duplication and gene loss for multi-copy genes (*chia*, *gaa*, *lyg*, *lyz* and *pgc*). One digestive enzyme gene *chia*, which is involved in breaking down chitin of insects, was found to be multi-copied with the copy number varying significantly among species (Fig. S1). We noticed that the common cuckoo (*Cuculus canorus*), whose diet is mostly composed of insects, possessed the highest gene copy number of *chia* (*n* = 4). Phylogenetic analysis showed that multiple events of gene duplication and gene loss have occurred in *chia* over the course of avian evolution (Fig. S1). Similarly, phylogenetic tree of the *lyz* genes showed that the *lyz* genes of birds formed two distinct clusters, of which one belonged to the conventional *lyz* and the other belonged to the Ca^2+^-binding *lyz* (Fig. S2). Notably, both the Peking duck (*Anas platyrhynchos*) and common cuckoo were found to have two intact *lyz* genes, and phylogenetic analysis suggested that the two copies in duck belonged separately to the conventional and Ca^2+^-binding type, whereas both copies from common cuckoo belonged to the Ca^2+^-binding group (Fig. S2). For the *lyg* gene, our phylogenetic tree showed that the *lyg* genes from birds were classified into three groups with high posterior probabilities (Fig. S3), being consistent with a previous study where three categories of *lyg* (*lygA*, *lygB* and *lygC*) were proposed (Irwin, 2014). The occurrence of all three types of *lyg* genes in the Chinese softshell turtle (*Pelodiscus sinensis* ) and Chinese alligator (*Alligator sinensis* ) suggested that the duplication of *lyg* predates the divergence between birds and reptiles (Irwin, 2014). Additionally, the Australaves clade containing nine species from Cariamiformes, Falconiformes, Psittaciformes and Passeriformes was found to possess the single copy *lygA* without the other two copies (*lygB* and *lygC*) (Fig. 1), thus, we speculated that *lygB* and *lygC* losses may have independently occurred in the common ancestor of Australaves after divergence from Coraciimorphae (Fig. 1 and Fig. S3). For the *gaa* gene, our phylogenetic tree showed that *gaa* genes of birds formed two distinct clusters (Fig. S4), which were denoted as *gaa i* and *gaa ii* by Kunita et al. (1997), who first discovered the two *gaa* copies in the Japanese quail (*Coturnix coturnix japonica*). The phylogenetic tree of *pgc* genes showed three distinct clusters: the basal cluster (*pgc*) included species from Galloanserae and Palaeognathae, where phylogenetic relationship agreed with the avian species tree (Prum et al., 2015) (Fig. S5); the other two clusters (*pgb1* and *pgb2*) contained all species from Neoaves. Additionally, most species such as the golden-collared manakin (*Manacus vitellinus*), Anna’s hummingbird (*Calypte anna*), and emperor penguin (*Aptenodytes forsteri*) possess two copies of *pgc* with one in each cluster (Fig. S5). Given that a *pgb* gene was identified in both chicken (*Gallus gallus*) and turkey (*Meleagris gallopavo*) (Castro et al., 2012), we speculated that the loss of *pgc* might have occurred in the common ancestor of Neoaves since their separation from Galloanserae, resulting in only species from Galloanserae and Palaeognathae harboring the *pgc* gene.

Gene family analyses on five datasets of multi-copy genes indicated that the global birth and death rate (*λ*) for all datasets was 0.001, and the global error rate was calculated to be 0.248. For each gene family, gene copies of all ancestral lineage nodes are shown in Fig. S6. The most recent common ancestor of the 48 bird species was estimated to have one *gaa*, two *pgc*, two *chia*, one *lyz*, and three *lyg* gene copies, respectively (Fig. S6). Notably, for the *chia* gene, reduction of copy number in lineages leading to the yellow-throated sandgrouse and white-tailed eagle, and copy number gains in the lineage leading to the common cuckoo were inferred to be rapidly evolved with *P* values <0.01 (Fig. S6). Moreover, an additional rapidly evolved branch with a reduction of *gaa* gene copies has occurred in the lineage leading to the white-tailed eagle (Fig. S6).

### Selection pressure analysis

To test whether specific codons of digestive enzyme genes have been subject to positive selection, we compared a pair of site models (M8 and M8a) and found that all genes except *agl*, *g6pc*, *gaa* and *gck*, have a significant better fit for the M8 model relative to the M8a model (Table 2), suggesting that a proportion of sites have undergone positive selection. The average *ω* values were estimated: 2.209 (hepatic *amy*), 2.639 (pancreatic *amy*), 1.501 (*cyp7a1*), 2.244 (*pnlip*), 1.464 (*pgb1*), 1.731 (*pgb2*), 3.747 (*chia*), 1.593 (*lygA*), 3.092 (*lygB*), 2.606 (*lygC*) and 1.743 (*lyz*). A total of 3, 5, 6, 15, 8, 9, 3, 5, 14, 3 and 3 codons, respectively, were separately identified to be under positive selection using the BEB approach with posterior probability ≥ 0.9 (Table 2). After combining with three other methods implemented in the Datamonkey webserver, sites identified by at least two of the four methods were regarded as candidates under positive selection. As a result, a total of 148 positively selected sites were identified, comprising of: 14 (hepatic *amy*), nine (pancreatic *amy*), 16 (*cyp7a1*), 40 (*pnlip*), 12 (*pgb1*), 19 (*pgb2*), four (*chia*), 11 (*lygA*), 11 (*lygB*), four (*lygC*), and eight (*lyz*) (Table S5). Furthermore, 97.97% (145/148) of the putative positively selected sites were predicted to have experienced radical changes in structural or biochemical properties at the amino acid level by TreeSAAP (Table S5), implying that these digestive enzymes play important roles in shaping avian dietary diversity.

**Table 2:** Likelihood ratio tests of site-specific models on avian digestive enzyme genes performed in PAML (Yang, 2007).

**Category**	**Gene**	**Site models**	**ln*L***^a^	***P*value**^b^	***ω* in M8**	**Positively selected sites**^c^
Carbohydrases	hepatic *amy*	M8	−8,835.49	0.0002^b^	2.209	4(0.92) 70(0.97) 477(0.99)
		M8a	−8,842.33			
	pancreatic *amy*	M8	−9,610.63	0.0001^b^	2.639	22(1.00) 23(0.97) 157(0.98) 367(0.96) 455(0.99)
		M8a	−9,617.84			
	*agl*	M8	−27,765.85	0.1195	1.154	none
		M8a	−27,767.07			
	*g6pc*	M8	−7,572.03	0.0852	1.550	none
		M8a	−7,573.51			
	*gaa i*	M8	−10,960.54	0.1796	1.439	none
		M8a	−10,961.44			
	*gaa ii*	M8	−8,355.26	0.4193	1.425	none
		M8a	−8,354.93			
	*gck*	M8	−4,081.65	0.9931	5.490	none
		M8a	−4,081.65			
Lipases	*cyp7a1*	M8	−12,177.26	0.0010^b^	1.501	3(0.98) 10(0.95) 30(0.96) 327(0.92) 392(0.97) 501(0.94)
		M8a	−12,182.67			
	*pnlip*	M8	−15,109.12	9.14E–28^b^	2.244	38(1.00) 66(1.00) 75(1.00) 76(0.95) 79(1.00) 80(1.00) 184(0.96) 250(0.91) 256(1.00) 312(1.00) 318(1.00) 340(1.00) 390(0.99) 402(0.99) 424(1.00)
		M8a	−15,168.75			
Proteases	*pgb1*	M8	−10,322.64	0.0002^b^	1.464	50(0.92) 133(0.96) 216(1.00) 220(0.97) 234(0.93) 248(0.91) 288(0.94) 343(0.94)
		M8a	−10,329.80			
	*pgb2*	M8	−9,220.00	5.17E-08^b^	1.731	108(0.99) 118(0.99) 197(0.95) 212(0.91) 253(1.00) 254(0.93) 261(1.00) 262(0.97) 273(0.95)
		M8a	−9,234.82			
Chitinase	*chia*	M8	−5,885.92	2.58E–06^b^	3.747	401(0.99) 402(1.00) 411(0.95)
		M8a	−5,896.97			
Lysozymes	*lygA*	M8	−5,992.18	2.17E–05^b^	1.593	11(0.99) 23(0.95) 24(0.94) 83(1.00) 131(1.00)
		M8a	−6,001.20			
	*lygB*	M8	−4,314.25	1.98E–26^b^	3.092	8(0.94) 10(1.00) 22(1.00) 33(0.99) 81(1.00) 83(1.00) 88(0.96) 91(1.00) 99(1.00) 103(0.95) 110(1.00) 128(1.00) 129(1.00) 132(0.98)
		M8a	−4,370.83			
	*lygC*	M8	−4,318.04	1.25E–07^b^	2.606	22(1.00) 39(1.00) 128(1.00)
		M8a	−4,332.01			
	*lyz*	M8	−4,100.09	0.0035^b^	1.743	31(0.97) 50(0.97) 58(0.92)
		M8a	−4,104.34			

**Notes.**

a The natural logarithm of likelihood value.

b *P* values denoted with two asterisks (**) when less than 0.01.

c Positively selected sites with posterior probabilities ≥ 0.9. Posterior probability values are shown in parentheses next to site numbers, which follow chicken genes.

**Figure 2:**
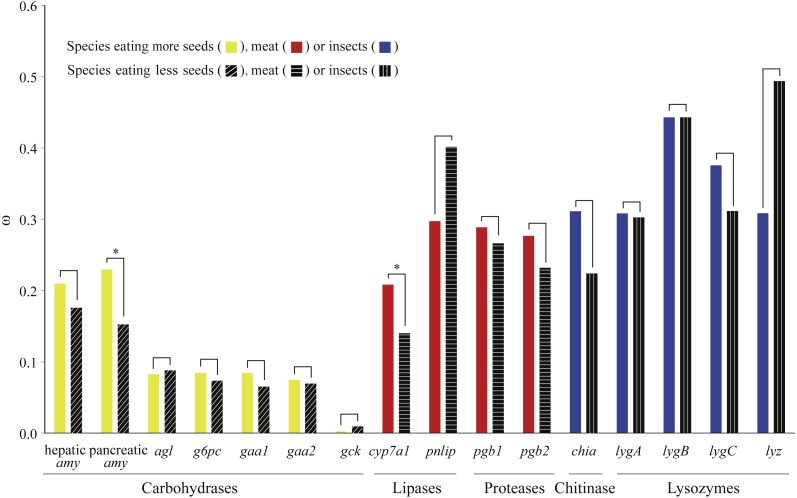
Differences in *ω* for each digestive enzyme gene between two groups of birds. For amylases, species were divided into two groups with contrasting seed ingestion; for carbohydrases (excluding the amylases), species were divided into two groups with contrasting ingestion of seeds, fruits and nectar; for lipases and proteases, species were divided into two groups with contrasting meat ingestion; for chitinases and lysozymes, species were divided into two groups with contrasting insect ingestion. *ω* for each group was estimated by the branch model implemented in PAML (Yang, 2007). An asterisk (*) indicates that *ω* estimated from the group with higher consumption of a particular food component is significantly greater than that from the other group with lower consumption.

We used a pair of branch models to test how selection pressure acted on digestive enzyme genes among bird species with different dietary habits. Through comparisons between nested models, we found that two genes (pancreatic *amy* and *cyp7a1*) derived from birds with specific dietary preferences have experienced adaptive evolution (Fig. 2 and Table S6). Specifically, pancreatic *amy* genes in species with a higher seed consumption (*ω* = 0.23) were shown to have a higher *ω* value than those in species with a lower seed consumption (*ω* = 0.15), with an FDR corrected *P* value at 0.01 (Table S6). Since the pancreatic amylase is important in digesting starch and is thus unlikely to undergo a relaxation from functional constraint, we suggested that a short burst of positive selection rather than a relaxation of selective pressure occurred on the pancreatic *amy* gene in birds consuming higher amount of seeds. The *cyp7a1* lipase gene was found to have evolved with a higher *ω* value in species with a higher meat consumption (*ω* = 0.21) than species with a lower meat consumption (*ω* = 0.14) (*P* = 0.01 after FDR adjustment) (Table S6). By contrast, genes coding for proteases and other enzymes involved in digesting insects did not show any signals of higher *ω* values in species with a higher meat or insect consumption (Fig. 2 and Table S6).

In addition, for these two genes (pancreatic *amy* and *cyp7a1*) that have undergone positive selection detected by branch models, positively selected sites identified by site models were labeled on specific branches where amino acid changes could lead to radical changes in structural or biochemical properties (Fig. S7). Notably, we found that pancreatic *amy* at the ancestral lineage of chicken and turkey experienced the same amino acid change (T455D) as the pigeon (*Columbia livia*) and the ancestral lineage of medium ground-finch (*Geospiza fortis*) and zebra finch (*Taeniopygia guttata*) (Fig. S7A). All five species mentioned above have a higher seed consumption (Fig. S7A); this is suggestive of convergent evolution. Similarly, an amino acid change (Q30R) in the *cyp7a1* gene was found to have occurred in ancestral or extant lineages with a higher meat consumption (Fig. S7B).

**Figure 3:**
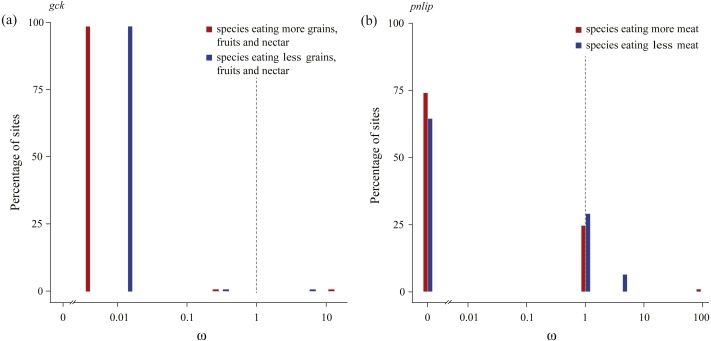
Patterns of natural selection on the carbohydrase gene *gck* and the lipase gene *pnlip*. All *ω* values were estimated by the best fitting models, with percentages of sites from each *ω* category plotted in blue (test branches) and red (reference branches). The gray vertical and dashed lines at *ω* = 1 indicates neutral evolution.

We subsequently tested in each gene whether the intensity of purifying selection is relaxed along test branches by the RELAX method implemented in HyPhy (Table 3). Our results showed that for the *gck* carbohydrase gene, when compared with species with a higher consumption of seeds, fruits, and nectar, test branches representing species with a lower consumption of those food items were shown to be significantly relaxed (Table 2). In addition, for the *pnlip* lipase gene, test branches representing species with a lower meat consumption were also shown to be significantly relaxed compared to those with a higher meat consumption (Table 3). Meanwhile, *ω* values of test branches estimated for both *gck* and *pnlip* were found to shift towards neutrality (*ω* = 1) (Fig. 3). The selection intensity parameter (*k*) in *gck* and *pnlip* was 0.75 and 0.59, with *P* values of 0.0049 and 0.0001, respectively (Table 3). However, the selection intensity of most digestive enzyme genes showed no significant differences between test and reference branches.

**Table 3:** Analyses on selection intensity for avian digestive enzyme genes conducted by RELAX (Wertheim et al., 2015).

**Category**	**Gene**	**Test branch**	**Reference branch**	**Models**	**ln***L*^a^	**2**Δ**(ln*L***)^b^	***P*value**^c^	***k***^d^
Carbohydrases	hepatic *amy* (17)	Below average seed consumption (12)	Above average seed consumption (5)	Null	−8,898.35	6.05	0.0139*	1.4047
				Alternative	−8,895.33			
	pancreatic *amy* (20)	Below average seed consumption (14)	Above average seed consumption (6)	Null	−9,791.24	0.76	0.3845	1.0685
				Alternative	−9,790.87			
	*agl* (46)	Below average consumption of grain, fruit and nectar (30)	Above average consumption of grain, fruit and nectar (16)	Null	−28,168.88	0.77	0.3807	0.9656
				Alternative	−28,168.49			
	*g6pc* (27)	Below average consumption of grain, fruit and nectar (18)	Above average consumption of grain, fruit and nectar (9)	Null	−7,639.34	0.01	0.9135	0.9907
				Alternative	−7,639.33			
	*gaa i* (25)	Below average consumption of grain, fruit and nectar (17)	Above average consumption of grain, fruit and nectar (8)	Null	−11,003.10	0.05	0.8257	0.9817
				Alternative	−11,003.08			
	*gaa ii* (14)	Below average consumption of grain, fruit and nectar (9)	Above average consumption of grain, fruit and nectar (5)	Null	−8,387.92	0.86	0.3537	0.9818
				Alternative	−8,387.49			
	*gck* (19)	Below average consumption of grain, fruit and nectar (12)	Above average consumption of grain, fruit and nectar (7)	Null	−3,975.70	7.93	0.0049^**^	**0.7504**
				Alternative	−3,971.73			
Lipases	*cyp7a1* (43)	Below average meat consumption (28)	Above average meat consumption (15)	Null	−12,473.08	8.75	0.0031^**^	1.4382
				Alternative	−12,468.70			
	*pnlip* (32)	Below average meat consumption (22)	Above average meat consumption (10)	Null	−15,888.46	16.44	0.0001^**^	**0.5897**
				Alternative	−15,880.24			
Proteases	*pgb1* (26)	Below average meat consumption (16)	Above average meat consumption (10)	Null	−10,536.91	6.55	0.0105*	9.1594
				Alternative	−10,533.64			
	*pgb2* (25)	Below average meat consumption (15)	Above average meat consumption (10)	Null	−9,387.55	0.67	0.4131	18.9967
				Alternative	−9,387.21			
Chitinase	*chia* (11)	Below average insect consumption (5)	Above average insect consumption (6)	Null	−5,922.95	0.001	0.9801	1.1284
				Alternative	−5,922.95			
Lysozymes	*lygA* (47)	Below average insect consumption (28)	Above average insect consumption (19)	Null	−6,170.60	0.55	0.4567	0.7211
				Alternative	−6,170.33			
	*lygB* (31)	Below average insect consumption (18)	Above average insect consumption (13)	Null	−4,549.53	0.11	0.7447	0.9469
				Alternative	−4,549.48			
	*lygC* (32)	Below average insect consumption (18)	Above average insect consumption (14)	Null	−4,400.81	6.86	0.0088^**^	2.0617
				Alternative	−4,397.38			
	*lyz* (19)	Below average insect consumption (13)	Above average insect consumption (6)	Null	−2,942.93	1.87	0.1711	5.8980
				Alternative	−2,941.99			

**Notes.**

a The natural logarithm of likelihood value.

b Twice the difference in ln *L* between two models compared.

c An asterisk (*) denotes *P* < 0.05, whiletwo asterisks (**) indicate *P* < 0.01.

d Significant *P* values with *k* < 1 or *k* > 1 indicate that selection strength is relaxed or intensified along the test branches, respectively.

In columns “Gene”, “Test branch” and “Reference branch”, numbers in parentheses represent total numbers of intact sequences. Two genes (gck and pnlip) were predicted to be under relaxed selection, which simultaneously require *P* < 0.05 and *k* < 1 (both are underlined and shown in bold).

### Functional divergence analysis

We next estimated functional divergence between clusters of four multi-copy genes (*gaa*, *lyz*, *lyg* and *pgc*), and the results are summarized in Table 4. Besides the comparison between Ca^2+^-binding *lyz* and conventional *lyz*, type-I functional divergence detecting altered functional constraints showed that the coefficients of type-I functional divergence (*θ*_*I*_) in any pairs of *gaa*, *lyg* and *pgc* were significantly greater than 0, suggestive of site-specific rate shift after gene duplication occurred in them (Table 4). Moreover, type-II functional divergence that detects radical changes in amino acid biochemical properties was inferred. Statistical significance of coefficients of type-II functional divergence (*θ*_*II*_) estimated between four gene pairs (*gaa i*/*gaa ii*, *lygA*/*lygB*, *lygA*/*lygC* and *pgb1*/*pgb2*) indicated that radical changes in amino acid properties might contribute to functional divergence (Table 4). To further identify the critical amino acid sites (CAASs) that might be responsible for functional divergence, the cutoff of posterior probability (Q_*k*_) >0.95 was used. We found that for six gene pairs *gaa i*/*gaa ii*, Ca^2+^-binding *lyz*/conventional *lyz*, *lygA*/*lygB*, *lygA*/*lygC, lygB*/*lygC* and *pgb1*/*pgb2*, a total of 20, 0, 36, 45, 5 and 34 CAASs were identified for type-I functional divergence, respectively, and a total of 190, 27, 63, 56, 16 and 99 CAASs, respectively, were identified for type-II functional divergence (Table 4). No CAASs were identified for type-I functional divergence between Ca^2+^-binding *lyz*/conventional *lyz*, whereas 27 sites were detected for type-II functional divergence (Table 4). Thus, we speculated that these 27 sites between clusters of *lyz* might serve as the main driver to facilitate the shift in evolutionary rate.

**Table 4:** Type-I and type-II functional divergences between sub-clusters of *gaa, lyz, lyg* and *pgc* in birds, estimated by Gu’s method (Gu & Vander Velden, 2002).

**Gene**	**Cluster1**	**Cluster2**	**Type-I**				**Type-II**			
			*θ*_*I*_ ± *SE*^a^	*LRT*^b^	*P* value^c^	Q_*k*_ >0.95^d^	*θ*_*II*_ ± *SE*^e^	*z*-score^f^	*P* value^c^	Q_*k*_ >0.95^d^
*gaa*	*gaa i*	*gaa ii*	0.60 ± 0.05	163.34	<0.01	20	0.27 ± 0.03	7.66	<0.01	190
*lyz*	Ca ^2+^-binding *lyz*	Conventional *lyz*	0.26 ± 0.16	2.59	0.108	0	0.14 ± 0.13	1.12	0.265	27
*lyg*	*lygA*	*lygB*	0.78 ± 0.05	272.87	<0.01	36	0.36 ± 0.10	3.53	<0.01	63
	*lygA*	*lygC*	0.85 ± 0.05	286.11	<0.01	45	0.33 ± 0.11	2.86	<0.01	56
	*lygB*	*lygC*	0.29 ± 0.04	44.52	<0.01	5	0.06 ± 0.12	0.51	0.612	16
*pgc*	*pgb1*	*pgb2*	0.57 ± 0.04	248.04	<0.01	34	0.23 ± 0.09	2.73	<0.01	99

**Notes.**

a The maximum likelihood estimate of functional divergence coefficient (*θ*_*I*_) with the standard error (SE).

b Maximum likelihood ratio estimated through type-I divergence analysis.

c The significance level estimated by chi-square test for type-I functional divergence and z-test for type-II functional divergence.

d Total number of sites with a posterior probability (*Q*_*k*_) > 0.95.

e The estimate of functional divergence coefficient (*θ*_*II*_) with standard error.

f The ratio of *θ*_*II*_ to SE.

### Ancestral state reconstruction

Based on the dietary data from Wilman et al. (2014), we conducted the reconstruction of ancestral states in dietary composition and the proportions of consuming seeds, meat, fruit or nectar, and invertebrates were chosen as four continuous characters. This reconstruction showed that the most recent common ancestor of extant birds tended to feed mainly on plant materials (e.g., seeds, fruits, nectar and other plant tissues) and invertebrates (e.g., insects) (Fig. S8). Furthermore, we found that meat gradually became the dominant dietary item in two distinct ancestral lineages: one leading to Aequorlitornithes, and the other leading to Accipitriformes (Fig. S8). In addition, ancestral lineages leading to several species consuming more fruit or nectar, such as Anna’s hummingbird, red-crested turaco (*Tauraco erythrolophus*), speckled mousebird (*Colius striatus*), golden-collared manakin, rhinoceros hornbill (*Buceros rhinoceros*) and white-throated tinamou (*Tinamus guttatus*), did not show a higher consumption of fruits or nectar (Fig. S8).

## Discussion

Birds have enormous taxonomic and ecological diversity. Understanding the diversity of dietary habits in birds is of great importance for evaluating their physiological adaptations to food resources and their surrounding environments over the course of avian evolution. Considering the irreplaceable roles of digestive enzymes in energy and nutrient uptake, we hypothesized that digestive enzyme genes may have experienced adaptive evolution among birds with different dietary habits. In this study, based on available genome sequences, we performed a large-scale evolutionary study on 16 digestive enzyme genes from 48 bird species with diverse diets to reconstruct the evolutionary history of digestive enzyme genes across the avian phylogeny.

### Gene loss and duplication

Among the 16 digestive enzyme genes, three (*chit1*, *lipf*, and salivary *amy*) were not found in all 48 avian genome sequences, and synteny analysis further indicated that these genes may be absent in the common ancestor of birds. For the 13 genes found in birds, the discovery that most birds possess one pancreatic *amy* and one hepatic *amy* is consistent with an earlier study by Benkel et al. (2005), who identified two distinct *amy* loci in chicken using probe hybridization, with one locus expressed in the pancreas and the second locus detected in the liver. For lipases, despite the absence of *lipf*, birds still have the pancreatic lipase PNLIP, which was regarded as the primary lipases for breaking down dietary fat molecules during digestion (Lowe, 1997). For the other undetected gene *chit1*, earlier reports suggested that in mammals, the firstly determined functional chitinase CHIT1 and the secondly discovered functional CHIA, belonging to the family 18 of glycosyl hydrolases, had the same chitinolytic activity and resulted from an earlier gene duplication event (Renkema et al., 1998; Boot et al., 2001). Since frogs were observed to have two copies of active chitinases, of which one copy was clustered with mammalian CHIA (Fujimoto et al., 2002), the gene duplication generating the two copies of chitinases occurred before the divergence of tetrapods accompanied by the development of the acidic stomach (Bussink et al., 2007). Consequently, the absence of *chit1* in birds may represent a subsequent loss after the duplication of the two chitinase copies. We speculated that the deficiency in physiological activity caused by these three undetected genes (salivary *amy*, *lipf* and *chit1*) might be compensated through other candidate genes with similar functions.

With regard to multi-copy digestive enzyme genes, the g-type lysozyme gene identified in birds is mostly triplicated, with three copies tandemly distributed at the same scaffold or chromosome, which were previously named *lygA*, *lygB* and *lygC* (Irwin, 2014). Through analysis on the reconstructed phylogenetic tree, we discovered that nine bird species from the clade Australaves only have *lygA* without both *lygB* and *lygC* (Fig. 1 and Fig. S3), suggesting that the losses of *lygB* and *lygC* have independently occurred in the ancestor of Australaves after divergence from Coraciimorphae. Phylogenetic relationships of both *lyz* and *gaa* (Figs. S2 and S4) showed two distinct gene clusters, which were consistent with earlier discoveries (Kunita et al., 1997; Irwin, Biegel & Stewart, 2011). Moreover, the evolution of avian pepsinogen gene (*pgc*) turned out to be complex and the *pgc* and *pgb1* genes we referred to here were respectively named as *pgc2* and *pgb* by Castro et al. (2012), who studied the evolution of pepsinogen *C* genes in vertebrates and found only one *pgb* copy in both chicken and turkey. However, by searching 48 avian genomes for *pgc* and reconstructing their phylogenetic relationships, we discovered that apart from some species (chicken, turkey, ostrich and duck) with a single *pgc* gene, most species possess two distinct *pgc* genes (*pgb1* and *pgb2*), with each type of *pgc* comprising an independent cluster (Fig. S5). Considering that all species from Neoaves lack one copy of *pgc*, it is suggested that the loss of *pgc* has occurred after the divergence of Galloanserae from Palaeognathae and before Neoaves split from Galloanserae.

Gene family analyses on five multi-copy genes indicated that gene copies of the most recent common ancestor of extant birds were estimated to have one *gaa*, two *pgc*, two *chia*, one *lyz*, and three *lyg* gene copies, and the *chia* gene had rapidly evolved in several lineages such as those leading to the sandgrouse, the white-tailed eagle and the common cuckoo (Fig. S6). Given that gene copy numbers of *chia* in mammals were suggested to be positively correlated with dietary insects (Janiak, Chaney & Tosi, 2017), we predicted that the four *chia* copies detected in the common cuckoo might be related to their specialized diet of insects.

### Positive selection

Positively selected sites were detected in all digestive enzyme genes except *agl*, *g6pc*, *gaa* and *gck*, and a total of 148 codons were identified to be under positive selection by at least two methods. Furthermore, adaptive evolution was further supported by evidence that a high percentage (97.97%) of the putative positively selected sites was under radical changes in structural or biochemical properties at the amino acid level, suggesting that the properties and functions of digestive enzymes might have been influenced by radical amino acid changes. Overall, positive selection on digestive enzymes may serve as the major evolutionary force driving the formation of diverse diets in birds.

By contrast, we did not detect any avian lineages showing an *ω* greater than one, a strong signature of positive selection. Instead, we found multiple lineages showing a significantly higher *ω*, suggesting a short burst of positive selection or a relaxation of selective pressure. The pancreatic *amy* encoding amylase, which plays an essential role in hydrolyzing dietary starch by acting on *α*-1,4-glycosidic bonds, was detected to have a higher *ω* value in species with a higher seed consumption (Fig. 2 and Table S6). We predicted that this signature was a short burst of positive selection rather than a relaxation of selective pressure, because the pancreatic amylase is important in digesting starch and is thus unlikely to undergo a relaxation from functional constraint. Indeed, previous experimental evidences have pointed that the activities of pancreatic amylase in six passerines were positively correlated with their dietary starches (Kohl et al., 2010; Kohl et al., 2011). Similar patterns of amylase activity associated with dietary carbohydrates were also found in other groups of animals, such as fruit flies (Hickey & Benkel, 1982), fishes (Hidalgo, Urea & Sanz, 1999), dogs (Axelsson et al., 2013). The higher *ω* value of the pancreatic *amy* detected in birds consuming a higher amount of seeds may indicate a higher efficiency in enzymatic hydrolysis of pancreatic amylase.

The carbohydrase gene *gck*, with no positively selected sites detected by the M8 model (Table 2), was found to be under relaxed selection in species consuming a lower amount of seeds, fruits and nectar, when compared with those with consuming a higher amount (Table 3). The enzyme glucokinase (GCK), encoded by gene *gck*, participates in the phosphorylation of glucose to glucose-6-phosphate, which is the first step of both glycolysis and glycogen synthesis, and plays a major role in the regulation of carbohydrate metabolism (Cardenas, 1995). Enzymatic assays showed that compared to fasted chickens, fed chickens were found to have significantly increased GCK activity in liver homogenates (Berradi et al., 2005). Our findings in *gck* implied that the bird species consuming more seeds, fruits, and nectar might have evolved a distinct digestive strategy to adapt to higher dietary carbohydrates and facilitate the absorption of glucose and glycolysis.

Lipases play key roles in catalyzing dietary triglyceride into monoglycerides and fatty acids. In this study we found that the *cyp7a1* lipase gene has a higher *ω* value in species eating more meat (Fig. 2), suggesting a short burst of positive selection. Lipase CYP7A1, belonging to the superfamily Cytochrome P450, is an oxidoreductase and can limit the rate of bile acid synthesis (Russell & Setchell, 1992). In animals, bile acids are mainly synthesized in the liver and function as surfactants that can emulsify dietary fats into micelles and keep them suspended before further digestion and absorption (Hofmann & Borgstroem, 1964). Another lipase gene, *pnlip*, which was found to be under relaxed selection in species eating less meat, has a function in the small intestine for hydrolyzation of dietary triacylglyceride (Hegele et al., 2001). The higher *ω* value of *cyp7a1* and the relatively intensified selection of *pnlip* detected in birds eating more meat indicate that lipases might be under greater selective pressure for digesting lipids. Furthermore, we found that one codon (site 10) located in the predicted helical transmembrane segment of *cyp7a1* was positively selected, the same codon was also found to be positively selected in cetaceans (Wang et al., 2016), implying that this site might be of functional importance for enzyme CYP7A1 in efficiently digesting lipids.

Previous enzymatic assays of one peptidase (APN) and two proteases (TRY and CTRC) in six passerines showed that activities of the three digestive enzymes were not positively correlated with the contents of dietary protein (Kohl et al., 2010). This finding was consistent with our evolutionary analysis on proteases and several enzymes involved in digesting insects, wherein we did not find significant differences in *ω* values among different bird lineages, considering the abundant protein contents in insects. We hypothesized that proteases and enzymes involved in digesting insects may have not generated significant differences in selective pressure among avian lineages at the sequence level, given that all birds require proteins to maintain their normal life activities and most birds prey on insects during their breeding seasons (Del Hoyo, Elliott & Sargatal, 1994).

### Ancestral state reconstruction

Reconstruction of ancestral states indicated that the most recent common ancestor of extant birds were omnivorous and mainly fed on invertebrates and plant materials (seeds, fruits, nectar, or other plant materials) (Fig. S8). However, several groups among living birds had evolved to be specialists feeding on a particular food, such as hoatzins and hummingbirds. Previous studies suggested that omnivory in birds acted as a macroevolutionary sink with higher extinction rates and lower speciation rates (Burin et al., 2016), and omnivorous birds could be favored at places or times with low abundance of a preferred resource or when resource availability was not highly predictable (Macarthur & Levins, 1967), while those birds with specific diets might only survive when food resources were sustainable and highly predictable (Karr, 1976). Considering the unstable climates in the late-Cretaceous that led to a mass extinction of most species (Longrich, Tokaryk & Field, 2011) and the subsequent relatively stable climates in the Cenozoic (Kissling et al., 2012), we speculated that omnivorous birds might have survived from the late-Cretaceous, and the stable and highly predictable resources in the following Cenozoic might function as the main evolutionary force driving dietary specialization. Coincided with our prediction that seeds were one of the ancestral food components in the most recent common ancestor of extant birds, fossil evidence also suggested that seeds were a key factor for the survival of edentulous grain-consuming birds through the late-Cretaceous mass extinction (Larson, Brown & Evans, 2016).

### Limitations

There are two limitations in this study. First, some inherent copies of digestive enzyme genes cannot be identified from the avian genomes and some coding sequences are not complete. For instance, with most coding sequences being incomplete or inherent copies being missing, only six intact *ctrc* (single-copy) and 12 intact *chia* (multi-copy) genes were identified from 48 genome sequences. Due to the incompleteness of some coding sequences, we only used intact or longer partial coding sequences for evolutionary analyses, which might have caused bias. Meanwhile, in order to evaluate the influence of avian genome information (assembly and annotation) on the number of gene copies obtained, we conducted gene family analyses which estimated the value of global error rate to be 0.248. Second, it is possible that the evaluation of food composition was not entirely accurate, given that the standardized and semiquantitative numeric information from the comprehensive avian diet database was converted from avian dietary data or verbal description. Therefore, high-quality genome sequences and more accurate dietary data will help understand the evolution of digestive enzymes and dietary diversification in birds in the future.

## Conclusions

We detected positively selected sites in most examined digestive enzyme genes, suggesting that different diets may have favored differences in catalytic capacities of these enzymes. The pancreatic *amy* and the *cyp7a1* lipase gene were found to have a higher *ω* in species consuming more seeds and meat, respectively, suggesting a short burst of positive selection on the corresponding lineages. The *gck* carbohydrase gene in species consuming less seeds, fruits, or nectar, and the *pnlip* lipase gene in species consuming less meat, were found to have undergone relaxed selection, suggesting that the two genes have become less important in the corresponding lineages. Together with our functional divergence and gene family analyses, our results document that multiple evolutionary processes have shaped the evolution of digestive enzymes in birds, and suggest that the evolutionary flexibility of these enzymes may have facilitated their dietary diversification.

##  Supplemental Information

10.7717/peerj.6840/supp-1 Figure S1 Phylogenetic relationships reconstructed based on identified avian *chia* genes The phylogenetic tree was generated by the Bayesian method. Nodes with posterior probabilities below 50% were collapsed. Note that *chia* genes used here include the partial sequences spanning from exon 4 to exon 10. For convenience, species names of 48 birds are abbreviated as follows: *AcaChl* (*Acanthisitta chloris*); *AnaPla* (*Anas platyrhynchos*); *ApaVit* (*Apaloderma vittatum*); *AptFor* (*Aptenodytes forsteri*); *BalReg* (*Balearica regulorum*); *BucRhi* (*Buceros rhinoceros*); *CalAnn* (*Calypte anna*); *CapCar* (*Caprimulgus carolinensis*); *CarCri* (*Cariama cristata*); *CatAur* (*Cathartes aura*); *ChaPel* (*Chaetura pelagica*); *ChaVoc* (*Charadrius vociferus*); *ChlMac* (*Chlamydotis macqueenii*); *ColLiv* (*Columba livia*); *ColStr* (*Colius striatus*); *CorBra* (*Corvus brachyrhynchos*); *CucCan* (*Cuculus canorus*); *EgrGar* (*Egretta garzetta*);* EurHel* (*Eurypyga helias*); *FalPer* (*Falco peregrinus*); *FulGla* (*Fulmarus glacialis*); *GalGal* (*Gallus gallus*)*; GavSte* (*Gavia stellata*);* GeoFor* (*Geospiza fortis*);* HalAlb* (*Haliaeetus albicilla*);* HalLeu* (*Haliaeetus leucocephalus*); *LepDis* (*Leptosomus discolor*); *ManVit* (*Manacus vitellinus*); *MelGal* (*Meleagris gallopavo*); *MelUnd* (*Melopsittacus undulatus*); *MerNub* (*Merops nubicus*); *MesUni* (*Mesitornis unicolor*); *NesNot* (*Nestor notabilis*); *NipNip* (*Nipponia nippon*); *OpiHoa* (*Opisthocomus hoazin*); *PelCri* (*Pelecanus crispus*); *PhaLep* (*Phaethon lepturus*); *PhaCar* (*Phalacrocorax carbo*); *PhoRub* (*Phoenicopterus ruber*); *PicPub* (*Picoides pubescens*); *PodCri* (*Podiceps cristatus*); *PteGut* (*Pterocles gutturalis*); *PygAde* (*Pygoscelis adeliae*); *StrCam* (*Struthio camelus*); *TaeGut* (*Taeniopygia guttata*); *TauEry* (*Tauraco erythrolophus*); *TinGut* (*Tinamus guttatus*); *TytAlb* (*Tyto alba*).

10.7717/peerj.6840/supp-2 Figure S2 Phylogenetic relationships reconstructed based on identified avian *lyz* genes Bayesian inference was applied to reconstruct the phylogenetic tree. Nodes with posterior probabilities below 50% were collapsed. The c-type lysozyme genes were classified into two groups: the Ca ^2+^-binding type and the conventional type. Species names are abbreviated as described in the legend of Fig. S1.

10.7717/peerj.6840/supp-3 Figure S3 Phylogenetic relationships reconstructed based on identified avian *lyg* genes The phylogenetic tree was generated by Bayesian methods. Nodes with posterior probabilities below 50% were collapsed. The g-type lysozyme genes identified in birds were clustered into three groups, and three groups were named as *lygA*, *lygB* and *lygC*, respectively, according to the nomenclature proposed by Irwin (2014) . Species names of birds are abbreviated as described in the legend of Fig. S1.

10.7717/peerj.6840/supp-4 Figure S4 Phylogenetic relationships reconstructed based on identified avian *gaa* genes The phylogenetic tree was generated by Bayesian methods. Nodes with posterior probabilities below 50% were collapsed. The *gaa* genes identified in birds were clustered into two distinct groups, which were separately named as *gaa i* and *gaa ii* by Kunita et al. (1997). Species names of birds are abbreviated as described in the legend of Fig. S1.

10.7717/peerj.6840/supp-5 Figure S5 Phylogenetic relationships reconstructed based on identified avian *pgc* genes The phylogenetic tree was generated by Bayesian methods. Nodes with posterior probabilities below 50% were collapsed. According to Castro et al. (2012) , we defined three clusters of avian pepsinogen *C* genes as *pgc*, *pgb1* and *pgb2*. Species names of birds are abbreviated as described in the legend of Fig. S1.

10.7717/peerj.6840/supp-6 Figure S6 Evolutionary changes of multi-copied gene numbers Gene numbers for ancestral and extant lineages were shown in black, with the numbers from left to right representing the estimated copy number of *gaa*, *pgc*, *chia*, *lyz* and *lyg*, whereas the numbers of expansion and contraction in gene family were indicated with red and blue, respectively. Rapidly evolved lineages were denoted with an asterisk (*) above the corresponding gene copy number and higher taxon names were shown at selected nodes.

10.7717/peerj.6840/supp-7 Figure S7 Graphic representation of radical amino acid changes in putative selected sites of the pancreatic* amy* and *cyp7a1* (A) Species eating more grains were indicated in bold; (B) Species eating more meat were shown in bold. Each color represents a unique codon site.

10.7717/peerj.6840/supp-8 Figure S8 Ancestral state reconstruction of avian dietary composition The pie chart shows indicated the percentage of dietary seeds (yellow), meat (red), insects (blue), fruits and nectar (orange) and other plant materials (blue). Higher taxon names were shown at selected nodes.

10.7717/peerj.6840/supp-9 Table S1 Food composition data of 48 birds*

10.7717/peerj.6840/supp-10 Table S2 Basic statistics for the assemblies of 48 avian species*

10.7717/peerj.6840/supp-11 Table S3 Specified avian branches for selection tests on the consumption of a particular dietary item

10.7717/peerj.6840/supp-12 Table S4 Synteny analysis on undetected *chit1*, *lipf* and salivary* amy* in 48 avian genomic sequences

10.7717/peerj.6840/supp-13 Table S5 Radical amino acid sites under positive selection detected by PAML, Datamonkey and TreeSAAP simultaneously

10.7717/peerj.6840/supp-14 Table S6 Likelihood ratio tests of selective pressures on avian digestive enzyme genes by branch models

10.7717/peerj.6840/supp-15 Dataset S1 Nucleotide sequences of 13 identified digestive enzyme genes, including pseudogenes, partial and intact genes

10.7717/peerj.6840/supp-16 Supplemental Material S1 Supplementary Material for Evolution of digestive enzymes and dietary diversification in birds

## Additional Information and Declarations

### Data Availability

The following information was supplied regarding data availability: The dataset supporting the conclusions of this article is included in the article and Dataset S1.

## References

[ref-1] Akaike H (1974). A new look at the statistical model identification. IEEE Transactions on Automatic Control.

[ref-2] Amano K, Ito E (1978). The action of lysozyme on partially deacetylated chitin. European Journal of Biochemistry.

[ref-3] Axelsson E, Ratnakumar A, Arendt ML, Maqbool K, Webster MT, Perloski M, Liberg O, Arnemo JM, Hedhammar A, Lindblad-Toh K (2013). The genomic signature of dog domestication reveals adaptation to a starch-rich diet. Nature.

[ref-4] Bao Y, Yang BZ, Dawson TL, Chen Y (1997). Isolation and nucleotide sequence of human liver glycogen debranching enzyme mRNA: identification of multiple tissue-specific isoforms. Gene.

[ref-5] Benkel BF, Nguyen T, Uno Y, De Leon FAP, Hickey DA (2005). Structural organization and chromosomal location of the chicken alpha-amylase gene family. Gene.

[ref-6] Berger LR, Weiser RS (1957). The beta-glucosaminidase activity of egg-white lysozyme. Biochimica et Biophysica Acta.

[ref-7] Berradi H, Taouis M, Cassy S, Rideau N (2005). Glucokinase in chicken (*Gallus gallus*). Partial cDNA cloning, immunodetection and activity determination. Comparative Biochemistry and Physiology Part B: Biochemistry and Molecular Biology.

[ref-8] Boot RG, Blommaart EF, Swart E, Ghauharali-van der Vlugt K, Bijl N, Moe C, Place A, Aerts JM (2001). Identification of a novel acidic mammalian chitinase distinct from chitotriosidase. Journal of Biological Chemistry.

[ref-9] Boot RG, Renkema GH, Strijland A, Van Zonneveld AJ, Aerts JMFG (1995). Cloning of a cDNA encoding chitotriosidase, a human chitinase produced by macrophages. Journal of Biological Chemistry.

[ref-10] Burin G, Kissling WD, Guimaraes PR, Şekercioglu CH, Quental TB (2016). Omnivory in birds is a macroevolutionary sink. Nature Communications.

[ref-11] Bussink AP, Speijer D, Aerts JM, Boot RG (2007). Evolution of mammalian chitinase(-like) members of family 18 glycosyl hydrolases. Genetics.

[ref-12] Cardenas ML (1995). Glucokinase: its regulation and role in liver metabolism.

[ref-13] Carriere F, Barrowman JA, Verger R, Rene L (1993). Secretion and contribution to lipolysis of gastric and pancreatic lipases during a test meal in humans. Gastroenterology.

[ref-14] Castro LF, Lopes-Marques M, Goncalves O, Wilson JM (2012). The evolution of pepsinogen C genes in vertebrates: duplication, loss and functional diversification. PLOS ONE.

[ref-15] Dalloul RA, Long JA, Zimin AV, Aslam L, Beal K, Blomberg L, Bouffard P, Burt DW, Crasta O, Crooijmans RPMA, Cooper K, Coulombe RA, De S, Delany ME, Dodgson JB, Dong JJ, Evans C, Frederickson KM, Flicek P, Florea L, Folkerts O, Groenen MAM, Harkins TT, Herrero J, Hoffmann S, Megens HJ, Jiang A, De Jong P, Kaiser P, Kim H, Kim KW, Kim S, Langenberger D, Lee MK, Lee T, Mane S, Marcais G, Marz M, McElroy AP, Modise T, Nefedov M, Notredame C, Paton IR, Payne WS, Pertea G, Prickett D, Puiu D, Qioa D, Raineri E, Ruffier M, Salzberg SL, Schatz MC, Scheuring C, Schmidt CJ, Schroeder S, Searle SMJ, Smith EJ, Smith J, Sonstegard TS, Stadler PF, Tafer H, Tu ZJ, Van Tassell CP, Vilella AJ, Williams KP, Yorke JA, Zhang LQ, Zhang HB, Zhang XJ, Zhang Y, Reed KM (2010). Multi-platform next-generation sequencing of the domestic turkey (*Meleagris gallopavo*): genome assembly and analysis. PLOS Biology.

[ref-16] De Bie T, Cristianini N, Demuth JP, Hahn MW (2006). CAFE: a computational tool for the study of gene family evolution. Bioinformatics.

[ref-17] Del Hoyo J, Elliott A, Sargatal J (1994). Handbook of the birds of the world.

[ref-18] Desnuelle P (1960). The enzymes.

[ref-19] Dunn BM (2001). Overview of pepsin-like aspartic peptidases. Current Protocols in Protein Science.

[ref-20] Fujimoto W, Suzuki M, Kimura K, Iwanaga T (2002). Cellular expression of the gut chitinase in the stomach of frogs Xenopus laevis and Rana catesbeiana. Biomedical Research-tokyo.

[ref-21] Gill FB (1995). Ornithology.

[ref-22] Gratecos D, Desnuelle P (1971). On a chymotrypsin C purified from autolyzed porcine pancreas. Biochemical and Biophysical Research Communications.

[ref-23] Gu X, Vander Velden K (2002). DIVERGE: phylogeny-based analysis for functional-structural divergence of a protein family. Bioinformatics.

[ref-24] Hahn MW, De Bie T, Stajich JE, Nguyen C, Cristianini N (2005). Estimating the tempo and mode of gene family evolution from comparative genomic data. Genome Research.

[ref-25] Hegele RA, Ramdath DD, Ban MR, Carruthers MN, Carrington CV, Cao H (2001). Polymorphisms in PNLIP, encoding pancreatic lipase, and associations with metabolic traits. Journal of Human Genetics.

[ref-26] Hickey DA, Benkel B (1982). Regulation of amylase activity in Drosophila melanogaster: effects of dietary carbohydrate. Biochemical Genetics.

[ref-27] Hidalgo MC, Urea E, Sanz A (1999). Comparative study of digestive enzymes in fish with different nutritional habits. Proteolytic and amylase activities. Aquaculture.

[ref-28] Hillier LW, Miller W, Birney E, Warren W, Hardison RC, Ponting CP, Bork P, Burt DW, Groenen MAM, Delany ME, Dodgson JB, Chinwalla AT, Cliften PF, Clifton SW, Delehaunty KD, Fronick C, Fulton RS, Graves TA, Kremitzki C, Layman D, Magrini V, McPherson JD, Miner TL, Minx P, Nash WE, Nhan MN, Nelson JO, Oddy LG, Pohl CS, Randall-Maher J, Smith SM, Wallis JW, Yang SP, Romanov MN, Rondelli CM, Paton B, Smith J, Morrice D, Daniels L, Tempest HG, Robertson L, Masabanda JS, Griffin DK, Vignal A, Fillon V, Jacobbson L, Kerje S, Andersson L, Crooijmans RPM, Aerts J, Van der Poel JJ, Ellegren H, Caldwell RB, Hubbard SJ, Grafham DV, Kierzek AM, McLaren SR, Overton IM, Arakawa H, Beattie KJ, Bezzubov Y, Boardman PE, Bonfield JK, Croning MDR, Davies RM, Francis MD, Humphray SJ, Scott CE, Taylor RG, Tickle C, Brown WRA, Rogers J, Buerstedde JM, Wilson SA, Stubbs L, Ovcharenko I, Gordon L, Lucas S, Miller MM, Inoko H, Shiina T, Kaufman J, Salomonsen J, Skjoedt K, Wong GKS, Wang J, Liu B, Wang J, Yu J, Yang HM, Nefedov M, Koriabine M, deJong PJ, Goodstadt L, Webber C, Dickens NJ, Letunic I, Suyama M, Torrents D, Mering Cvon, Zdobnov EM, Makova K, Nekrutenko A, Elnitski L, Eswara P, King DC, Yang S, Tyekucheva S, Radakrishnan A, Harris RS, Chiaromonte F, Taylor J, He JB, Rijnkels M, Griffiths-Jones S, Ureta-Vidal A, Hoffman MM, Severin J, Searle SMJ, Law AS, Speed D, Waddington D, Cheng Z, Tuzun E, Eichler E, Bao ZR, Flicek P, Shteynberg DD, Brent MR, Bye JM, Huckle EJ, Chatterji S, Dewey C, Pachter L, Kouranov A, Mourelatos Z, Hatzigeorgiou AG, Paterson AH, Ivarie R, Brandstrom M, Axelsson E, Backstrom N, Berlin S, Webster MT, Pourquie O, Reymond A, Ucla C, Antonarakis SE, Long MY, Emerson JJ, Betran E, Dupanloup I, Kaessmann H, Hinrichs AS, Bejerano G, Furey TS, Harte RA, Raney B, Siepel A, Kent WJ, Haussler D, Eyras E, Castelo R, Abril JF, Castellano S, Camara F, Parra G, Guigo R, Bourque G, Tesler G, Pevzner PA, Smit A, Fulton LA, Mardis ER, Wilson RK (2004). Sequence and comparative analysis of the chicken genome provide unique perspectives on vertebrate evolution. Nature.

[ref-29] Hofmann AF, Borgstroem B (1964). The intraluminal phase of fat digestion in man: the lipid content of the micellar and oil phases of intestinal content obtained during fat digestion and absorption. The Journal of Clinical Investigation.

[ref-30] Hornbuckle WE, Simpson KW, Tennant BC (2008). Gastrointestinal function.

[ref-31] Houde A, Kademi A, Leblanc D (2004). Lipases and their industrial applications: an overview. Applied Biochemistry and Biotechnology.

[ref-32] Irwin DM (2014). Evolution of the vertebrate goose-type lysozyme gene family. BMC Evolutionary Biology.

[ref-33] Irwin DM, Biegel JM, Stewart C (2011). Evolution of the mammalian lysozyme gene family. BMC Evolutionary Biology.

[ref-34] Irwin DM, Gong ZM (2003). Molecular evolution of vertebrate goose-yype lysozyme genes. Journal of Molecular Evolution.

[ref-35] Janiak MC, Chaney ME, Tosi AJ (2017). Evolution of acidic mammalian chitinase genes (*CHIA*) is related to body mass and insectivory in primates. Molecular Biology and Evolution.

[ref-36] Jarvis ED, Mirarab S, Aberer AJ, Li B, Houde P, Li C, Ho SYW, Faircloth BC, Nabholz B, Howard JT, Suh A, Weber CC, Da Fonseca RR, Li JW, Zhang F, Li H, Zhou L, Narula N, Liu L, Ganapathy G, Boussau B, Bayzid MS, Zavidovych V, Subramanian S, Gabaldon T, Capella-Gutierrez S, Huerta-Cepas J, Rekepalli B, Munch K, Schierup M, Lindow B, Warren WC, Ray D, Green RE, Bruford MW, Zhan XJ, Dixon A, Li SB, Li N, Huang YH, Derryberry EP, Bertelsen MF, Sheldon FH, Brumfield RT, Mello CV, Lovell PV, Wirthlin M, Schneider MPC, Prosdocimi F, Samaniego JA, Velazquez AMV, Alfaro-Nunez A, Campos PF, Petersen B, Sicheritz-Ponten T, Pas A, Bailey T, Scofield P, Bunce M, Lambert DM, Zhou Q, Perelman P, Driskell AC, Shapiro B, Xiong ZJ, Zeng YL, Liu SP, Li ZY, Liu BH, Wu K, Xiao J, Yinqi X, Zheng QM, Zhang Y, Yang HM, Wang J, Smeds L, Rheindt FE, Braun M, Fjeldsa J, Orlando L, Barker FK, Jonsson KA, Johnson W, Koepfli KP, O’Brien S, Haussler D, Ryder OA, Rahbek C, Willerslev E, Graves GR, Glenn TC, McCormack J, Burt D, Ellegren H, Alstrom P, Edwards SV, Stamatakis A, Mindell DP, Cracraft J, Braun EL, Warnow T, Jun W, Gilbert MTP, Zhang GJ (2014). Whole-genome analyses resolve early branches in the tree of life of modern birds. Science.

[ref-37] Jetton TL, Liang Y, Pettepher CC, Zimmerman EC, Cox FG, Horvath K, Matschinsky FM, Magnuson MA (1994). Analysis of upstream glucokinase promoter activity in transgenic mice and identification of glucokinase in rare neuroendocrine cells in the brain and gut. Journal of Biological Chemistry.

[ref-38] Karasov WH, Martinez del Rio C, Caviedes-Vidal E (2011). Ecological physiology of diet and digestive systems. Annual Review of Physiology.

[ref-39] Karr JR (1976). Within- and between-habitat avian diversity in African and neotropical lowland habitats. Ecological Monographs.

[ref-40] Kissling WD, Eiserhardt WL, Baker WJ, Borchsenius F, Couvreur TLP, Balslev H, Svenning J (2012). Cenozoic imprints on the phylogenetic structure of palm species assemblages worldwide. Proceedings of the National Academy of Sciences of the United States of America.

[ref-41] Kissling WD, Sekercioglu CH, Jetz W (2012). Bird dietary guild richness across latitudes, environments and biogeographic regions. Global Ecology and Biogeography.

[ref-42] Kohl KD, Brzek P, Caviedes-Vidal E, Karasov WH (2010). Matching between dietary preferences and digestive capacity in passerine birds. Integrative and Comparative Biology.

[ref-43] Kohl KD, Brzek P, Caviedes-Vidal E, Karasov WH (2011). Pancreatic and intestinal carbohydrases are matched to dietary starch level in wild passerine birds. Physiological and Biochemical Zoology.

[ref-44] Kornegay JR, Schilling JW, Wilson AC (1994). Molecular adaptation of a leaf-eating bird: stomach lysozyme of the hoatzin. Molecular Biology and Evolution.

[ref-45] Kramer KJ, Muthukrishnan S (1997). Insect chitinases: molecular biology and potential use as biopesticides. Insect Biochemistry and Molecular Biology.

[ref-46] Kunita R, Nakabayashi O, Wu JY, Hagiwara Y, Mizutani M, Pennybacker M, Chen Y, Kikuchi T (1997). Molecular cloning of acid *α*-glucosidase cDNA of Japanese quail (*Coturnix coturnix japonica*) and the lack of its mRNA in acid maltase deficient quails. Biochimica et Biophysica Acta.

[ref-47] Larson DW, Brown CM, Evans DC (2016). Dental disparity and ecological stability in bird-like dinosaurs prior to the end-Cretaceous mass extinction. Current Biology.

[ref-48] Levey DJ, Place AR, Rey PJ, Martinez del Rio C (1999). An experimental test of dietary enzyme modulation in pine warblers *Dendroica pinus*. Physiological and Biochemical Zoology.

[ref-49] Lindsay GJH (1984). Adsorption of rainbow trout (*Salmo gairdneri*) gastric lysozymes and chitinase by cellulose and chitin. Aquaculture.

[ref-50] Liu Y, He GM, Xu HH, Han XQ, Jones G, Rossiter SJ, Zhang SY (2014). Adaptive functional diversification of lysozyme in insectivorous bats. Molecular Biology and Evolution.

[ref-51] Longrich NR, Tokaryk T, Field DJ (2011). Mass extinction of birds at the Cretaceous–Paleogene (K–Pg) boundary. Proceedings of the National Academy of Sciences of the United States of America.

[ref-52] Lopez-Palomo V, Martinez-Victoria E, Yago MD, Lupiani MJ, Manas M (1997). Regulation by diet of the pancreas enzyme content of suckling goats. Archives of Physiology and Biochemistry.

[ref-53] Lowe ME (1997). Structure and function of pancreatic lipase and colipase. Annual Review of Nutrition.

[ref-54] Macarthur RH, Levins R (1967). The limiting similarity, convergence, and divergence of coexisting species. The American Naturalist.

[ref-55] Maddison WP, Maddison DR (2018). http://www.mesquiteproject.org.

[ref-56] Matschinsky FM, Ellerman JE (1968). Metabolism of glucose in the islets of langerhans. Journal of Biological Chemistry.

[ref-57] Martinez del Rio C, Baker HG, Baker I (1992). Ecological and evolutionary implications of digestive processes—bird preferences and the sugar constituents of floral nectar and fruit pulp. Cellular and Molecular Life Sciences.

[ref-58] McKenzie HA (1996). Alpha-lactalbumins and lysozymes.

[ref-59] Myant NB, Mitropoulos KA (1977). Cholesterol 7 alpha-hydroxylase. Journal of Lipid Research.

[ref-60] Nile CJ, Townes CL, Michailidis G, Hirst BH, Hall J (2004). Identification of chicken lysozyme g2 and its expression in the intestine. Cellular and Molecular Life Sciences.

[ref-61] Nordlie RC, Sukalski KA (1985). The Enzymes of Biological Membranes.

[ref-62] Nylander JAA (2004). MrModeltest v2 Program distributed by the author.

[ref-63] Pan C, Lei K, Chen H, Ward JM, Chou JY (1998). Ontogeny of the murine glucose-6-phosphatase system. Archives of Biochemistry and Biophysics.

[ref-64] Pond SLK, Frost SDW (2005). Datamonkey: rapid detection of selective pressure on individual sites of codon alignments. Bioinformatics.

[ref-65] Pond SLK, Frost SDW, Muse SV (2005). HyPhy: hypothesis testing using phylogenies. Bioinformatics.

[ref-66] Prum RO, Berv JS, Dornburg A, Field DJ, Townsend JP, Lemmon EM, Lemmon AR (2015). A comprehensive phylogeny of birds (Aves) using targeted next-generation DNA sequencing. Nature.

[ref-67] Rawlings ND, Barrett AJ (1993). Evolutionary families of peptidases. The Biochemical Journal.

[ref-68] Renkema GH, Boot RG, Au FL, Donker-Koopman WE, Strijland A, Muijsers AO, Hrebicek M, Aerts JMFG (1998). Chitotriosidase, a chitinase, and the 39-kDa human cartilage glycoprotein, a chitin-binding lectin, are homologues of family 18 glycosyl hydrolases secreted by human macrophages. European Journal of Biochemistry.

[ref-69] Ronquist F, Teslenko M, Van der Mark P, Ayres DL, Darling A, Hohna S, Larget B, Liu L, Suchard MA, Huelsenbeck JP (2012). MrBayes 3.2: efficient bayesian phylogenetic inference and model choice across a large model space. Systematic Biology.

[ref-70] Russell DW, Setchell KDR (1992). Bile-acid biosynthesis. Biochemistry.

[ref-71] Schondube JE, Herreram LG, Martinez del Rio C (2001). Diet and the evolution of digestion and renal function in phyllostomid bats. Zoology.

[ref-72] Sitrin MD (2014). The gastrointestinal system.

[ref-73] Sogaard M, Abe J, Svensson B (1993). *α*-amylases: structure and function. Carbohydrate Polymers.

[ref-74] Stewart CB, Schilling JW, Wilson AC (1987). Adaptive evolution in the stomach lysozymes of foregut fermenters. Nature.

[ref-75] Stewart CB, Wilson AC (1987). Sequence convergence and functional adaptation of stomach lysozymes from foregut fermenters. Cold Spring Harbor Symposia on Quantitative Biology.

[ref-76] Suarez RK, Welch KC (2011). The sugar oxidation cascade: aerial refueling in hummingbirds and nectar bats. The Journal of Experimental Biology.

[ref-77] Swaminathan N, Radhakrishnan AN (1965). Distribution of alpha-glucosidase activities in human & monkey small intestine. Indian Journal of Biochemistry.

[ref-78] Swanson KW, Irwin DM, Wilson AC (1991). Stomach lysozyme gene of the langur monkey - tests for convergence and positive selection. Journal of Molecular Evolution.

[ref-79] Swanson WJ, Nielsen R, Yang QF (2003). Pervasive adaptive evolution in mammalian fertilization proteins. Molecular Biology and Evolution.

[ref-80] Taggart RT, Cass LG, Mohandas T, Derby P, Barr PJ, Pals G, Bell GI (1989). Human pepsinogen C (progastricsin). Isolation of cDNA clones, localization to chromosome 6, and sequence homology with pepsinogen A. Journal of Biological Chemistry.

[ref-81] Vallim TQD, Tarling EJ, Edwards PA (2013). Pleiotropic roles of bile acids in metabolism. Cell Metabolism.

[ref-82] Walker DG, Rao S (1964). The role of glucokinase in the phosphorylation of glucose by rat liver. Biochemical Journal.

[ref-83] Wang ZF, Xu SX, Du KX, Huang F, Chen Z, Zhou KY, Ren WH, Yang G (2016). Evolution of digestive enzymes and RNASE1 provides insights into dietary switch of cetaceans. Molecular Biology and Evolution.

[ref-84] Wang K, Zhao HB (2015). Birds generally carry a small repertoire of bitter taste receptor genes. Genome Biology and Evolution.

[ref-85] Warren WC, Clayton DF, Ellegren H, Arnold AP, Hillier LW, Kunstner A, Searle S, White S, Vilella AJ, Fairley S, Heger A, Kong LS, Ponting CP, Jarvis ED, Mello CV, Minx P, Lovell P, Velho TAF, Ferris M, Balakrishnan CN, Sinha S, Blatti C, London SE, Li Y, Lin YC, George J, Sweedler J, Southey B, Gunaratne P, Watson M, Nam K, Backstrom N, Smeds L, Nabholz B, Itoh Y, Whitney O, Pfenning AR, Howard J, Voelker M, Skinner BM, Griffin DK, Ye L, McLaren WM, Flicek P, Quesada V, Velasco G, Lopez-Otin C, Puente XS, Olender T, Lancet D, Smit AFA, Hubley R, Konkel MK, Walker JA, Batzer MA, Gu WJ, Pollock DD, Chen L, Cheng Z, Eichler EE, Stapley J, Slate J, Ekblom R, Birkhead T, Burke T, Burt D, Scharff C, Adam I, Richard H, Sultan M, Soldatov A, Lehrach H, Edwards SV, Yang SP, Li XC, Graves T, Fulton L, Nelson J, Chinwalla A, Hou SF, Mardis ER, Wilson RK (2010). The Genome of a Songbird. Nature.

[ref-86] Wertheim JO, Murrell B, Smith MD, Pond SLK, Scheffler K (2015). RELAX: detecting relaxed selection in a phylogenetic framework. Molecular Biology and Evolution.

[ref-87] Whitcomb DC, Lowe ME (2007). Human pancreatic digestive enzymes. Digestive Diseases and Sciences.

[ref-88] Wilcox PE (1970). Chymotrypsinogens-chymotrypsins. Methods in Enzymology.

[ref-89] Wilman H, Belmaker J, Simpson JE, Rosa CDLa, Rivadeneira MM, Jetz W (2014). EltonTraits 1.0: species-level foraging attributes of the world’s birds and mammals. Ecology.

[ref-90] Yang ZH (2007). PAML 4: phylogenetic analysis by maximum likelihood. Molecular Biology and Evolution.

[ref-91] Zhang GJ, Li C, Li QY, Li B, Larkin DM, Lee C, Storz JF, Antunes A, Greenwold MJ, Meredith RW, Odeen A, Cui J, Zhou Q, Xu LH, Pan HL, Wang ZJ, Jin LJ, Zhang P, Hu HF, Yang W, Hu J, Xiao J, Yang ZK, Liu Y, Xie QL, Yu H, Lian JM, Wen P, Zhang F, Li H, Zeng YL, Xiong ZJ, Liu SP, Zhou L, Huang ZY, An N, Wang J, Zheng QM, Xiong YQ, Wang GB, Wang B, Wang JJ, Fan Y, Da Fonseca RR, Alfaro-Nunez A, Schubert M, Orlando L, Mourier T, Howard JT, Ganapathy G, Pfenning A, Whitney O, Rivas MV, Hara E, Smith J, Farre M, Narayan J, Slavov G, Romanov MN, Borges R, Machado JP, Khan I, Springer MS, Gatesy J, Hoffmann FG, Opazo JC, Hastad O, Sawyer RH, Kim H, Kim KW, Kim HJ, Cho S, Li N, Huang YH, Bruford MW, Zhan XJ, Dixon A, Bertelsen MF, Derryberry E, Warren W, Wilson RK, Li SB, Ray DA, Green RE, O’Brien SJ, Griffin D, Johnson WE, Haussler D, Ryder OA, Willerslev E, Graves GR, Alstrom P, Fjeldsa J, Mindell DP, Edwards SV, Braun EL, Rahbek C, Burt DW, Houde P, Zhang Y, Yang HM, Wang J, Jarvis ED, Gilbert MTP, Wang J (2014). Comparative genomics reveals insights into avian genome evolution and adaptation. Science.

