# Supplementary figures and images for: Evolution of digestive enzymes and dietary diversification in birds
Source: PeerJ. 2019 Apr 25;7:e6840. doi: 10.7717/peerj.6840 (PMC6487185; doi:10.7717/peerj.6840)

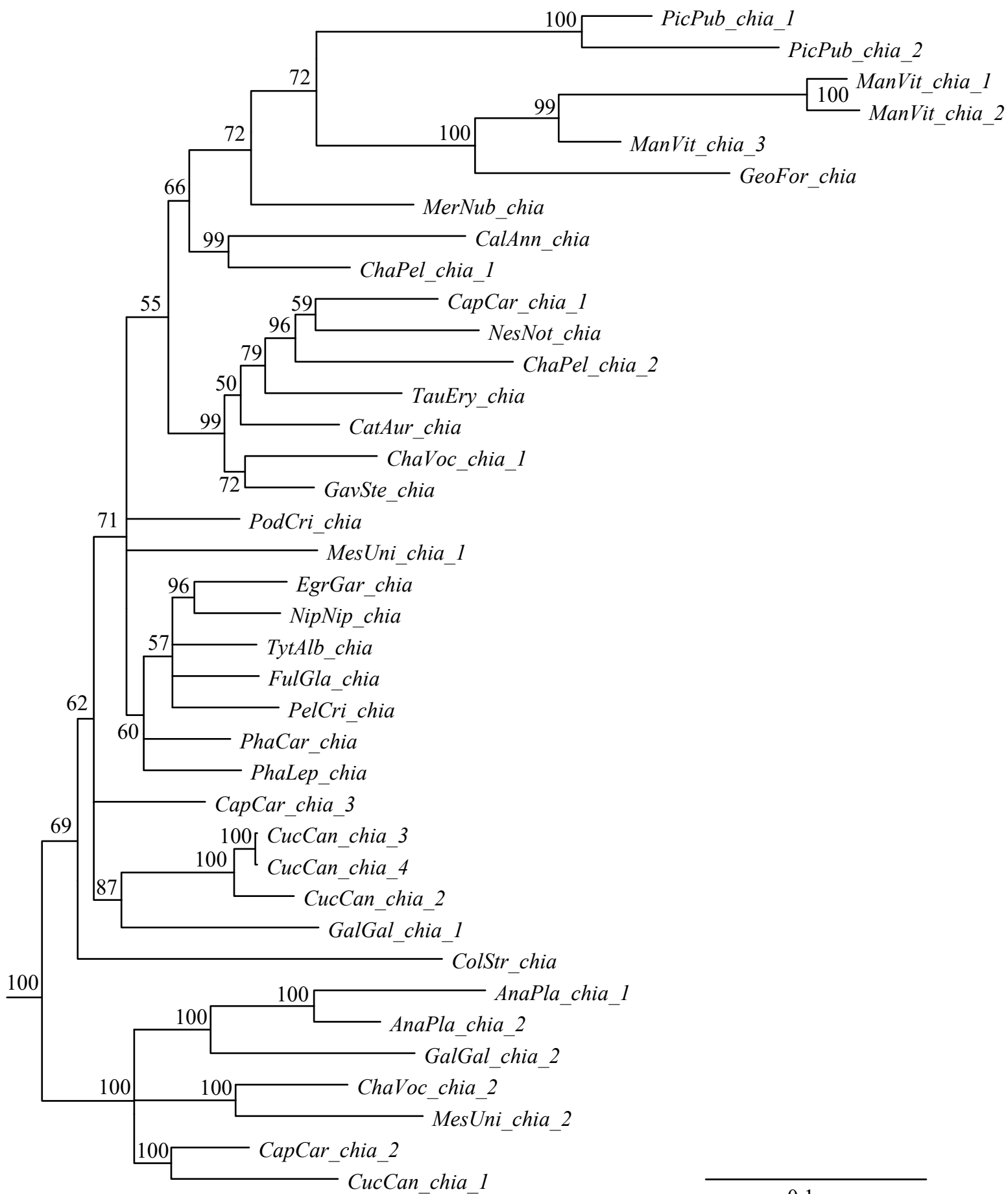

Supplement: Figure S1 — The phylogenetic tree was generated by the Bayesian method. Nodes with posterior probabilities below 50% were collapsed. Note that chia genes used here include the partial sequences spanning from exon 4 to exon 10. For convenience, species names of 48 birds are abbreviated as follows: AcaChl (Acanthisitta chloris); AnaPla (Anas platyrhynchos); ApaVit (Apaloderma vittatum); AptFor (Aptenodytes forsteri); BalReg (Balearica regulorum); BucRhi (Buceros rhinoceros); CalAnn (Calypte anna); CapCar (Caprimulgus carolinensis); CarCri (Cariama cristata); CatAur (Cathartes aura); ChaPel (Chaetura pelagica); ChaVoc (Charadrius vociferus); ChlMac (Chlamydotis macqueenii); ColLiv (Columba livia); ColStr (Colius striatus); CorBra (Corvus brachyrhynchos); CucCan (Cuculus canorus); EgrGar (Egretta garzetta); EurHel (Eurypyga helias); FalPer (Falco peregrinus); FulGla (Fulmarus glacialis); GalGal (Gallus gallus); GavSte (Gavia stellata); GeoFor (Geospiza fortis); HalAlb (Haliaeetus albicilla); HalLeu (Haliaeetus leucocephalus); LepDis (Leptosomus discolor); ManVit (Manacus vitellinus); MelGal (Meleagris gallopavo); MelUnd (Melopsittacus undulatus); MerNub (Merops nubicus); MesUni (Mesitornis unicolor); NesNot (Nestor notabilis); NipNip (Nipponia nippon); OpiHoa (Opisthocomus hoazin); PelCri (Pelecanus crispus); PhaLep (Phaethon lepturus); PhaCar (Phalacrocorax carbo); PhoRub (Phoenicopterus ruber); PicPub (Picoides pubescens); PodCri (Podiceps cristatus); PteGut (Pterocles gutturalis); PygAde (Pygoscelis adeliae); StrCam (Struthio camelus); TaeGut (Taeniopygia guttata); TauEry (Tauraco erythrolophus); TinGut (Tinamus guttatus); TytAlb (Tyto alba). [file peerj-07-6840-s001.pdf]

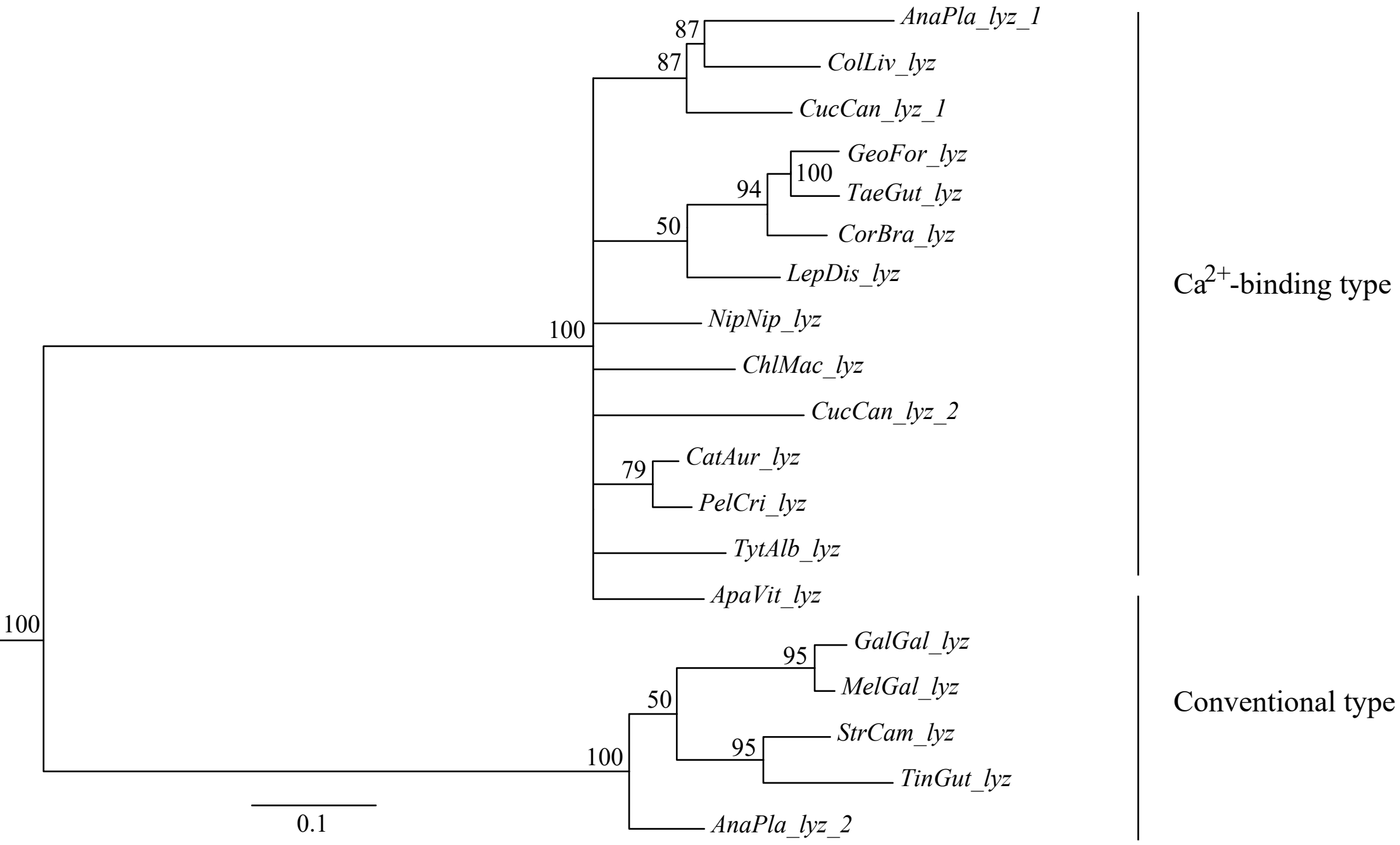

Supplement: Figure S2 — Bayesian inference was applied to reconstruct the phylogenetic tree. Nodes with posterior probabilities below 50% were collapsed. The c-type lysozyme genes were classified into two groups: the Ca 2+-binding type and the conventional type. Species names are abbreviated as described in the legend of Fig. S1. [file peerj-07-6840-s002.pdf]

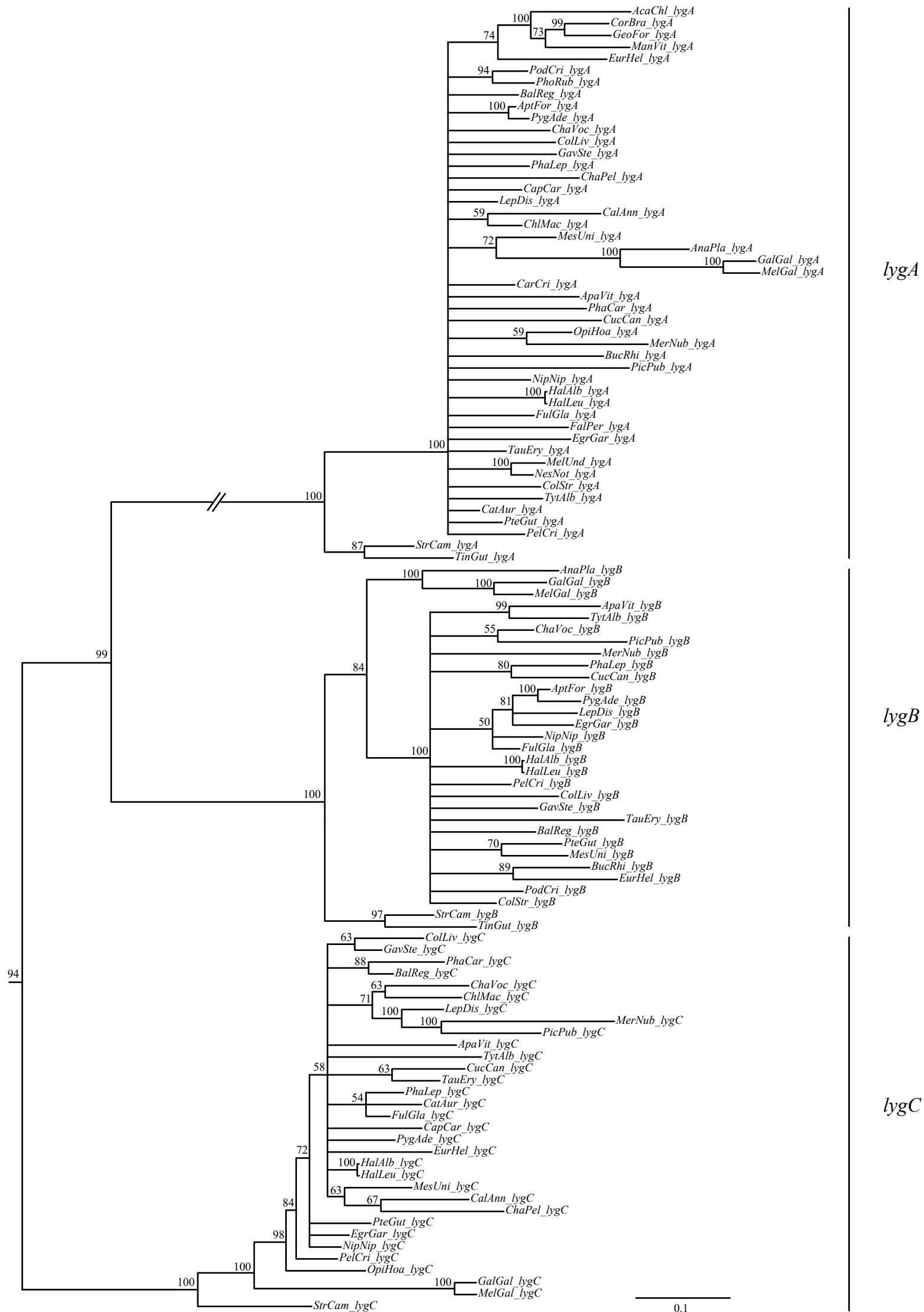

Supplement: Figure S3 — The phylogenetic tree was generated by Bayesian methods. Nodes with posterior probabilities below 50% were collapsed. The g-type lysozyme genes identified in birds were clustered into three groups, and three groups were named as lygA, lygB and lygC, respectively, according to the nomenclature proposed by Irwin (2014) . Species names of birds are abbreviated as described in the legend of Fig. S1. [file peerj-07-6840-s003.pdf]

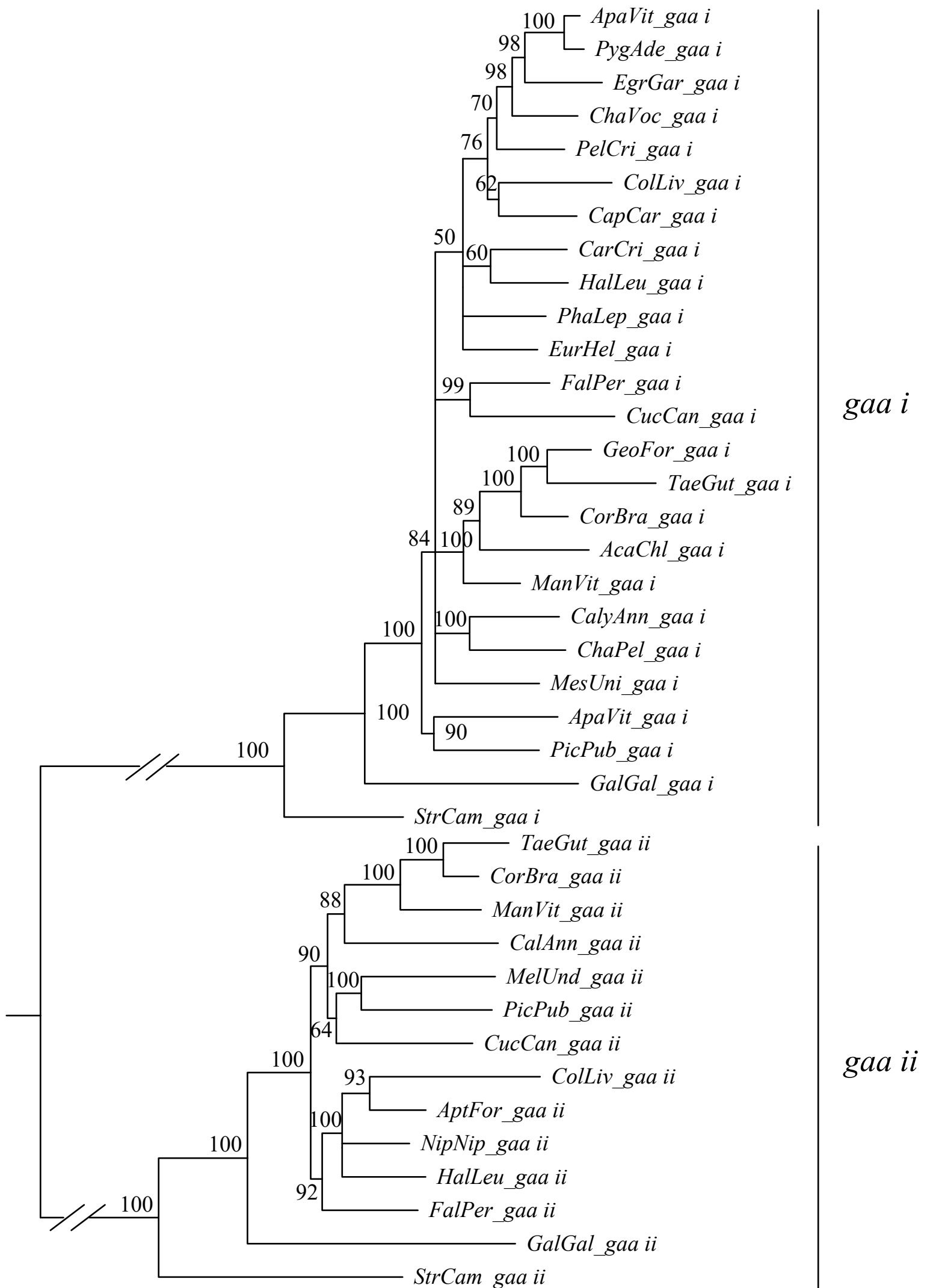

0.1

Supplement: Figure S4 — The phylogenetic tree was generated by Bayesian methods. Nodes with posterior probabilities below 50% were collapsed. The gaa genes identified in birds were clustered into two distinct groups, which were separately named as gaa i and gaa ii by Kunita et al. (1997). Species names of birds are abbreviated as described in the legend of Fig. S1. [file peerj-07-6840-s004.pdf]

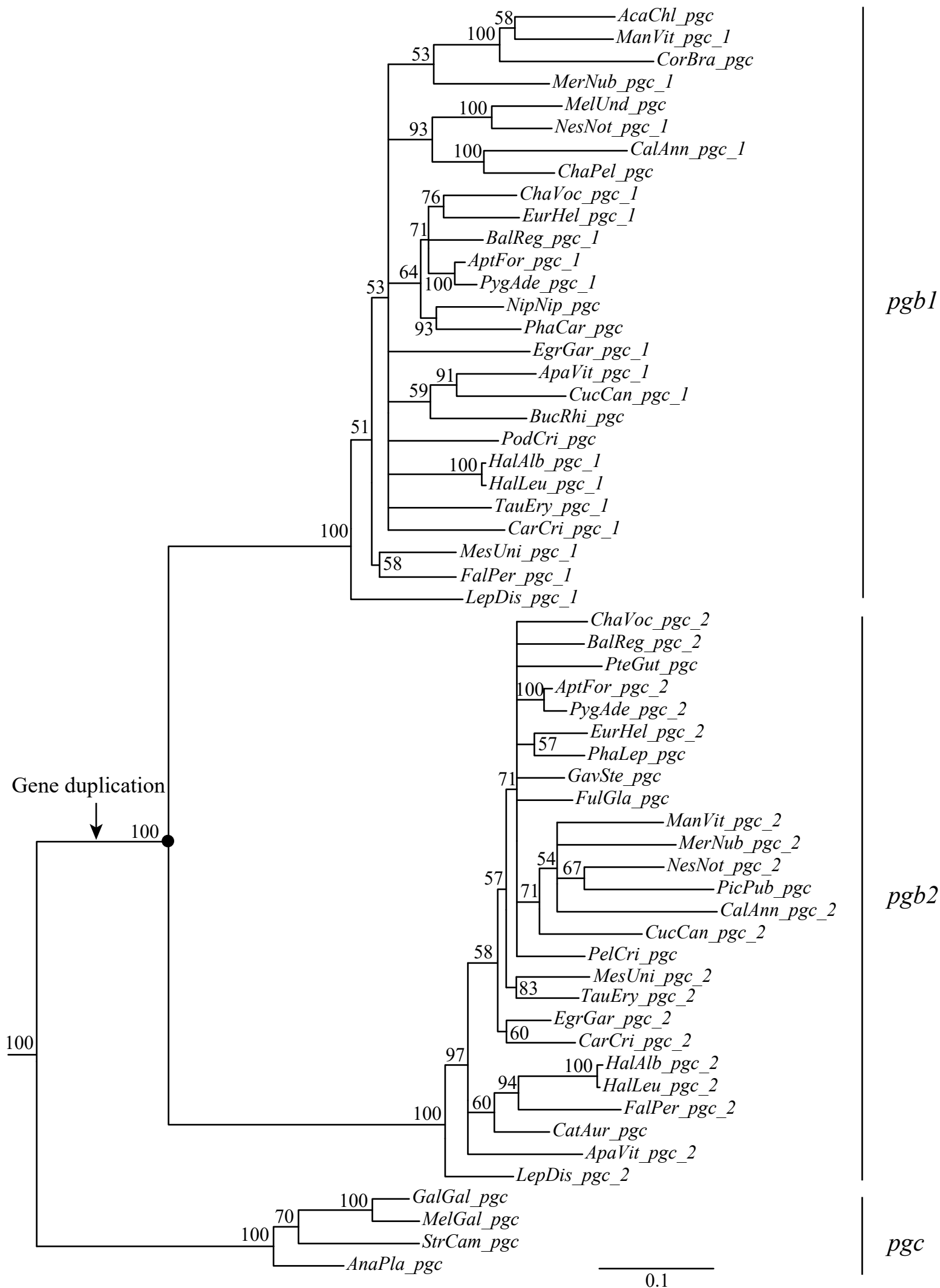

Supplement: Figure S5 — The phylogenetic tree was generated by Bayesian methods. Nodes with posterior probabilities below 50% were collapsed. According to Castro et al. (2012) , we defined three clusters of avian pepsinogen C genes as pgc, pgb1 and pgb2. Species names of birds are abbreviated as described in the legend of Fig. S1. [file peerj-07-6840-s005.pdf]

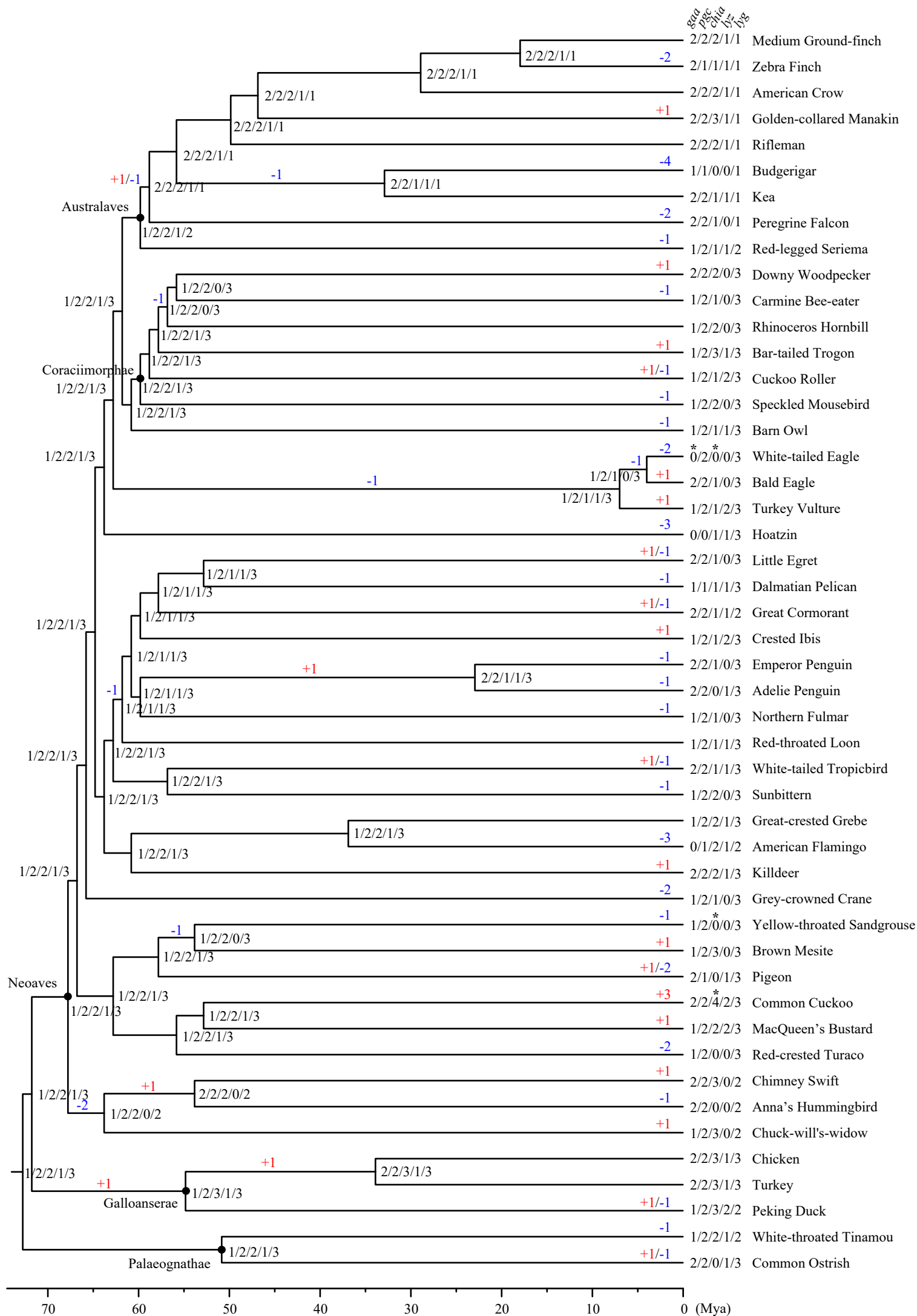

Supplement: Figure S6 — Gene numbers for ancestral and extant lineages were shown in black, with the numbers from left to right representing the estimated copy number of gaa, pgc, chia, lyz and lyg, whereas the numbers of expansion and contraction in gene family were indicated with red and blue, respectively. Rapidly evolved lineages were denoted with an asterisk (*) above the corresponding gene copy number and higher taxon names were shown at selected nodes. [file peerj-07-6840-s006.pdf]

# A pancreatic amy

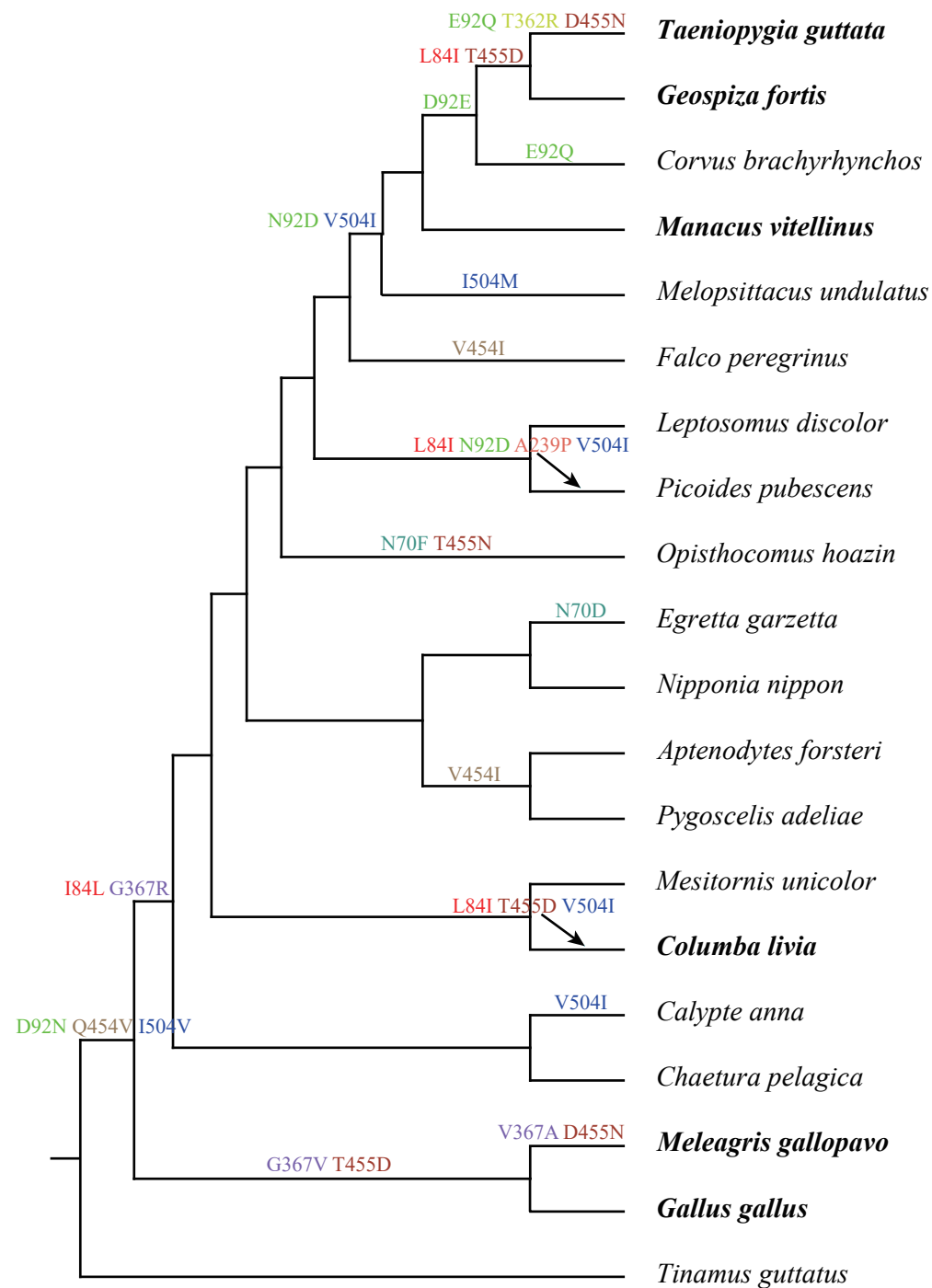

# B cyp7a1

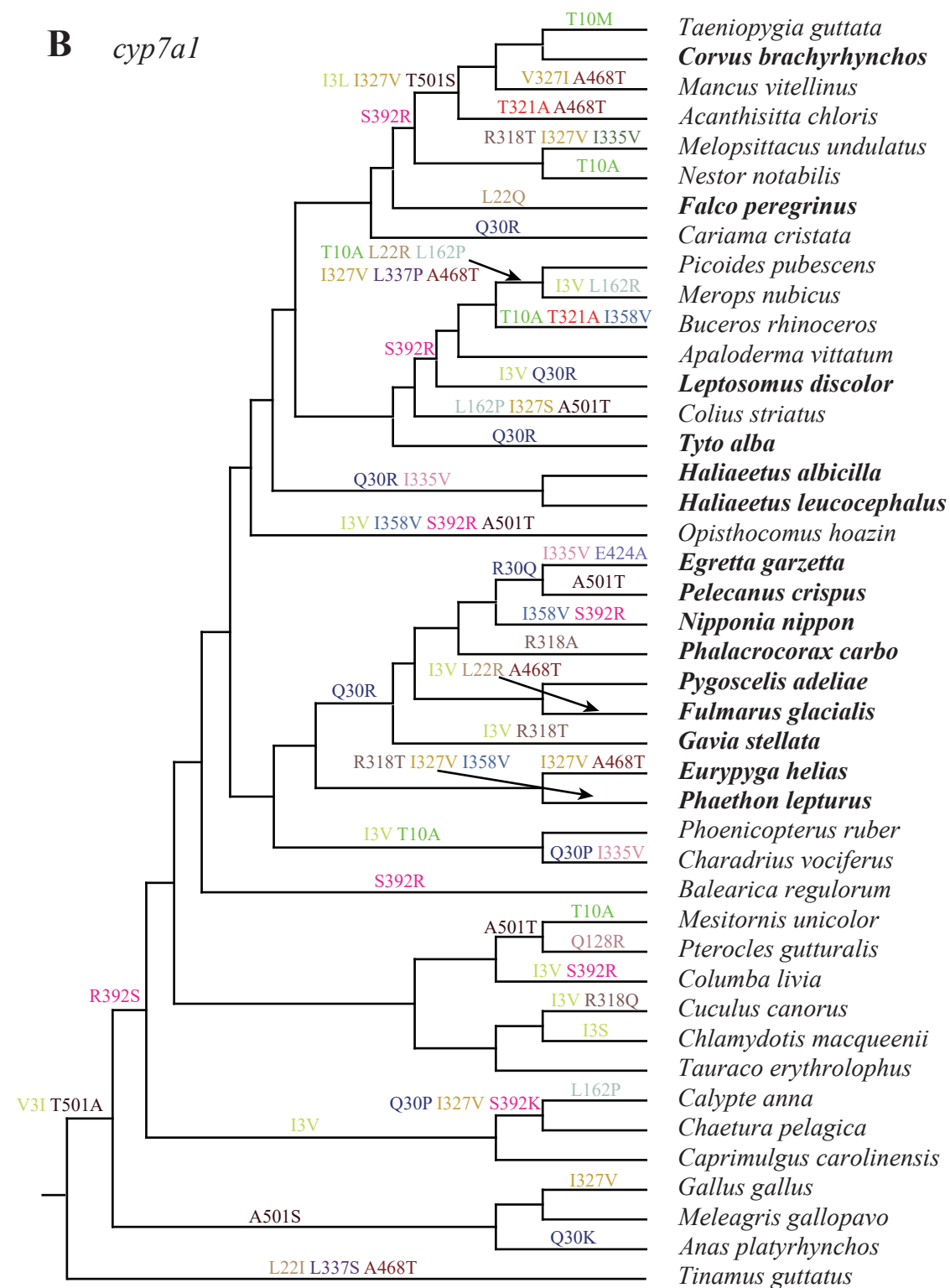

Supplement: Figure S7 — (A) Species eating more grains were indicated in bold; (B) Species eating more meat were shown in bold. Each color represents a unique codon site. [file peerj-07-6840-s007.pdf]

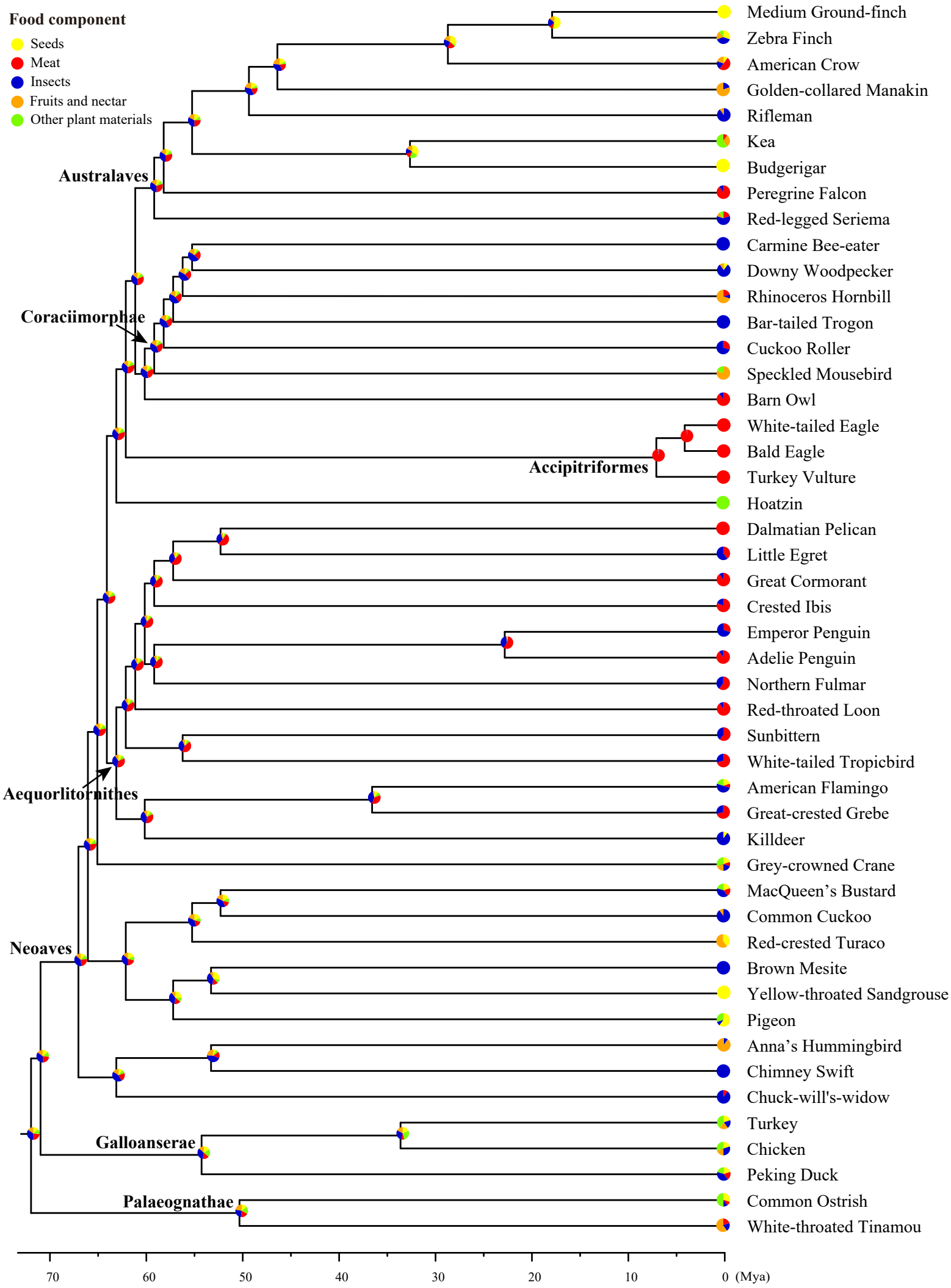

Supplement: Figure S8 — The pie chart shows indicated the percentage of dietary seeds (yellow), meat (red), insects (blue), fruits and nectar (orange) and other plant materials (blue). Higher taxon names were shown at selected nodes. [file peerj-07-6840-s008.pdf]
