# Supplementary material for: Evolution of digestive enzymes and dietary diversification in birds
Source: PeerJ. 2019 Apr 25;7:e6840. doi: 10.7717/peerj.6840 (PMC6487185; doi:10.7717/peerj.6840)
Supplement: Table S1 [file peerj-07-6840-s009.docx]

| **Order** | **Common name** | **Species name** | **Inv^a^** | **End^b^** | **Ect^c^** | **Fish^d^** | **Vun^e^** | **Sca^f^** | **Fru^g^** | **Nec^h^** | **Seed^i^** | **PlaO^j^** |
| --- | --- | --- | --- | --- | --- | --- | --- | --- | --- | --- | --- | --- |
| Accipitriformes | Turkey vulture | *Cathartes aura* | 0 | 0 | 0 | 0 | 0 | 100 | 0 | 0 | 0 | 0 |
| Accipitriformes | White-tailed eagle | *Haliaeetus albicilla* | 0 | 30 | 0 | 40 | 0 | 30 | 0 | 0 | 0 | 0 |
| Accipitriformes | Bald eagle | *Haliaeetus leucocephalus* | 0 | 30 | 20 | 30 | 0 | 20 | 0 | 0 | 0 | 0 |
| Anseriformes | Peking duck | *Anas platyrhynchos* | 40 | 0 | 10 | 10 | 0 | 0 | 0 | 0 | 20 | 20 |
| Apodiformes | Anna’s hummingbird | *Calypte anna* | 10 | 0 | 0 | 0 | 0 | 0 | 0 | 90 | 0 | 0 |
| Apodiformes | Chimney swift | *Chaetura pelagica* | 100 | 0 | 0 | 0 | 0 | 0 | 0 | 0 | 0 | 0 |
| Bucerotiformes | Rhinoceros hornbill | *Buceros rhinoceros* | 10 | 10 | 10 | 0 | 0 | 0 | 70 | 0 | 0 | 0 |
| Caprimulgiformes | Chuck-will’s-widow | *Caprimulgus carolinensis* | 90 | 10 | 0 | 0 | 0 | 0 | 0 | 0 | 0 | 0 |
| Cariamiformes | Red-legged seriema | *Cariama cristata* | 60 | 10 | 10 | 0 | 0 | 0 | 10 | 0 | 0 | 10 |
| Charadriiformes | Killdeer | *Charadrius vociferus* | 90 | 0 | 0 | 0 | 0 | 0 | 0 | 0 | 10 | 0 |
| Ciconiiformes | Crested ibis | *Nipponia nippon* | 20 | 0 | 10 | 70 | 0 | 0 | 0 | 0 | 0 | 0 |
| Ciconiiformes | Yellow-throated sandgrouse | *Pterocles gutturalis* | 0 | 0 | 0 | 0 | 0 | 0 | 0 | 0 | 100 | 0 |
| Coliiformes | Speckled mousebird | *Colius striatus* | 0 | 0 | 0 | 0 | 0 | 0 | 70 | 10 | 0 | 20 |
| Columbiformes | Domestic pigeon | *Columba livia* | 10 | 0 | 0 | 0 | 0 | 0 | 0 | 0 | 60 | 30 |
| Coraciiformes | Carmine bee-eater | *Merops nubicus* | 100 | 0 | 0 | 0 | 0 | 0 | 0 | 0 | 0 | 0 |
| Cuculiformes | Common cuckoo | *Cuculus canorus* | 90 | 0 | 0 | 0 | 0 | 0 | 10 | 0 | 0 | 0 |
| Falconiformes | Peregrine falcon | *Falco peregrinus* | 10 | 80 | 10 | 0 | 0 | 0 | 0 | 0 | 0 | 0 |
| Galliformes | Chicken | *Gallus gallus* | 30 | 0 | 0 | 0 | 0 | 0 | 20 | 0 | 20 | 30 |
| Galliformes | Turkey | *Meleagris gallopavo* | 20 | 0 | 0 | 0 | 0 | 0 | 20 | 0 | 20 | 40 |
| Gaviiformes | Red-throated loon | *Gavia stellata* | 10 | 0 | 0 | 90 | 0 | 0 | 0 | 0 | 0 | 0 |
| Gruiformes | Grey crowned-crane | *Balearica regulorum* | 40 | 0 | 20 | 0 | 0 | 0 | 0 | 0 | 20 | 20 |
| Gruiformes | Macqueen’s bustard | *Chlamydotis macqueenii* | 20 | 0 | 10 | 0 | 0 | 0 | 20 | 0 | 20 | 30 |
| Gruiformes | Sunbittern | *Eurypyga helias* | 40 | 0 | 30 | 30 | 0 | 0 | 0 | 0 | 0 | 0 |
| Gruiformes | Brown mesite | *Mesitornis unicolor* | 100 | 0 | 0 | 0 | 0 | 0 | 0 | 0 | 0 | 0 |
| Leptosomiformes | Cuckoo roller | *Leptosomus discolor* | 70 | 0 | 30 | 0 | 0 | 0 | 0 | 0 | 0 | 0 |
| Musophagiformes | Red-crested turaco | *Tauraco erythrolophus* | 0 | 0 | 0 | 0 | 0 | 0 | 60 | 0 | 40 | 0 |
| Opisthocomiformes | Hoatzin | *Opisthocomus hoazin* | 0 | 0 | 0 | 0 | 0 | 0 | 0 | 0 | 0 | 100 |
| Passeriformes | Rifleman | *Acanthisitta chloris* | 90 | 0 | 0 | 0 | 0 | 0 | 10 | 0 | 0 | 0 |
| Passeriformes | American crow | *Corvus brachyrhynchos* | 20 | 10 | 10 | 10 | 0 | 20 | 20 | 0 | 10 | 0 |
| Passeriformes | Medium ground-finch | *Geospiza fortis* | 40 | 0 | 0 | 0 | 0 | 0 | 20 | 0 | 30 | 10 |
| Passeriformes | Golden-collared manakin | *Manacus vitellinus* | 20 | 0 | 0 | 0 | 0 | 0 | 80 | 0 | 0 | 0 |
| Passeriformes | Zebra finch | *Taeniopygia guttata* | 0 | 0 | 0 | 0 | 0 | 0 | 0 | 0 | 100 | 0 |
| Pelecaniformes | Little egret | *Egretta garzetta* | 60 | 0 | 20 | 20 | 0 | 0 | 0 | 0 | 0 | 0 |
| Pelecaniformes | Dalmatian pelican | *Pelecanus crispus* | 0 | 0 | 0 | 100 | 0 | 0 | 0 | 0 | 0 | 0 |
| Pelecaniformes | White-tailed tropicbird | *Phaethon lepturus* | 30 | 0 | 0 | 70 | 0 | 0 | 0 | 0 | 0 | 0 |
| Pelecaniformes | Great cormorant | *Phalacrocorax carbo* | 10 | 0 | 10 | 80 | 0 | 0 | 0 | 0 | 0 | 0 |
| Phoenicopteriformes | American flamingo | *Phoenicopterus ruber* | 50 | 0 | 0 | 10 | 0 | 0 | 0 | 0 | 20 | 20 |
| Piciformes | Downy woodpecker | *Picoides pubescens* | 80 | 0 | 0 | 0 | 0 | 0 | 10 | 0 | 10 | 0 |
| Podicipediformes | Great crested grebe | *Podiceps cristatus* | 30 | 0 | 10 | 60 | 0 | 0 | 0 | 0 | 0 | 0 |
| Procellariiformes | Northern fulmar | *Fulmarus glacialis* | 40 | 0 | 0 | 60 | 0 | 0 | 0 | 0 | 0 | 0 |
| Psittaciformes | Budgerigar | *Melopsittacus undulatus* | 0 | 0 | 0 | 0 | 0 | 0 | 0 | 0 | 100 | 0 |
| Psittaciformes | Kea | *Nestor notabilis* | 0 | 0 | 0 | 0 | 0 | 10 | 30 | 0 | 0 | 60 |
| Sphenisciformes | Emperor penguin | *Aptenodytes forsteri* | 10 | 0 | 0 | 90 | 0 | 0 | 0 | 0 | 0 | 0 |
| Sphenisciformes | Adeliae penguin | *Pygoscelis adeliae* | 70 | 0 | 0 | 30 | 0 | 0 | 0 | 0 | 0 | 0 |
| Strigiformes | Barn owl | *Tyto alba* | 10 | 80 | 10 | 0 | 0 | 0 | 0 | 0 | 0 | 0 |
| Struthioniformes | African ostrich | *Struthio camelus* | 10 | 0 | 0 | 0 | 10 | 0 | 0 | 0 | 30 | 50 |
| Tinamiformes | White-throated tinamou | *Tinamus guttatus* | 20 | 0 | 0 | 0 | 20 | 0 | 60 | 0 | 0 | 0 |
| Trogoniformes | Bar-tailed trogon | *Apaloderma vittatum* | 100 | 0 | 0 | 0 | 0 | 0 | 0 | 0 | 0 | 0 |

^*^ Data source was derived from “EltonTraits 1.0: Species-level foraging attributes of the world’s birds and mammals,” by Wilman et al., 2014, *Ecology* 95: 2027. For analysis on pancreatic *amy* and hepatic *amy*, values in column “Seed” were selected for caculating the average seed consumption. For analysis on *agl*, *g6pc*, *gaa* and *gck*, values in columns “Seed”, “Fru” and “Nec” were selected for caculating the average consumption of seeds, fruits and nectar. For analysis on *cyp7a1*, *pnlip* and *pgc*, values in columns “End”, “Ect”, “Fish”, “Vun” and “Sca” were selected for caculating the average meat consumption. For analysis on *chia*, *lyz* and *lyg*, values in columns “Inv” were selected for caculating the average insect consumption.

^a^ Percentage of invertebrates-general, aquatic invertebrates, shrimp, krill, squid, crustacaeans, molluscs, cephalapod, polychaetes, gastropods, orthoptera, terrestrial invertebrates, ground insects, insect larvae, worms, orthopterans and flying insects.

^b^ Percentage of mammals and birds.

^c^ Percentage of reptiles, snakes, amphibians and salamanders

^d^ Percentage of fish.

^e^ Percentage of vertebrates-general or unknown.

^f^ Percentage of scavenge, garbage, offal, carcasses, trawlers and carrrion.

^g^ Percentage of fruit and drupes.

^h^ Percentage of nectar, pollen, plant exudates and gums.

^i^ Percentage of seed, maize, nuts, spores, wheat and grains.

^j^ Percentage of other plant material, grass, ground vegetation, seedlings, weeds, lichen, moss, small plants, reeds, cultivated crops, forbs, vegetables, fungi, roots, tubers, legumes, bulbs, leaves, above ground vegetation, twigs, bark, shrubs, herbs, shoots, aquatic vegetation and aquatic plants.
