# Supplementary material for: Evolution of digestive enzymes and dietary diversification in birds
Source: PeerJ. 2019 Apr 25;7:e6840. doi: 10.7717/peerj.6840 (PMC6487185; doi:10.7717/peerj.6840)
Supplement: Table S2 [file peerj-07-6840-s010.docx]

| **Order** | **Common name** | | **Species name** | **Sequence Depth** | **contig/scaffold N50** | **NCBI accessions** | **Ensemble accessions** |
| --- | --- | --- | --- | --- | --- | --- | --- |
| **Published (Sanger; turkey done with illumina+454)** | | | | | | | |
| Galliformes | Chicken | | *Gallus gallus* | 7X | 36K/7.07M | PRJNA13342 | Gallus_gallus4.0 |
| Passeriformes | Zebra finch | | *Taeniopygia guttata* | 6X | 39K/10M | PRJNA17289 | taeGut3.2.4 |
| Galliformes | Turkey | | *Meleagris gallopavo* | 17X | 12.6K/1.5M | PRJNA42129 | UMD2 |
| **High-coverage genomes** | | | | | | | |
| Psittaciformes | Budgerigar | | *Melopsittacus undulatus* | 160X | 55K/10.6M | PRJEB1588 | BGI_melUnd_0.0 |
| Passeriformes | Medium ground-finch | | *Geospiza fortis* | 115X | 30K/5.2M | PRJNA156703 | BGI_geoFor_0.0 |
| Apodiformes | Anna’s hummingbird | | *Calypte anna* | 110X | 23K/4M | PRJNA212866 | BGI_calAnn_0.0 |
| Passeriformes | Golden-collared manakin | | *Manacus vitellinus* | 110X | 34K/2.5M | PRJNA212872 | BGI_manVit_0.0 |
| Falconiformes | Peregrine falcon | | *Falco peregrinus* | 105X | 28K/3.9M | PRJNA159791 | BGI_falPer_0.0 |
| Ciconiiformes | Crested ibis | | *Nipponia nippon* | 105X | 22K/5.4M | PRJNA232572 | BGI_nipNip_0.0 |
| Piciformes | Downy woodpecker | | *Picoides pubescens* | 105X | 20K/2M | PRJNA212874 | BGI_picPub_0.0 |
| Apodiformes | Chimney swift | | *Chaetura pelagica* | 103X | 27K/3.8M | PRJNA210808 | BGI_chaPel_0.0 |
| Charadriiformes | Killdeer | | *Charadrius vociferus* | 100X | 32K/3.6M | PRJNA212867 | BGI_chaVoc_0.0 |
| Cuculiformes | Common cuckoo | | *Cuculus canorus* | 100X | 31K/3M | PRJNA212870 | BGI_cucCan_0.0 |
| Opisthocomiformes | Hoatzin | | *Opisthocomus hoazin* | 100X | 24K/2.9M | PRJNA212873 | BGI_ophHoa_0.0 |
| Tinamiformes | White-throated tinamou | | *Tinamus guttatus* | 100X | 24K/242K | PRJNA212876 | BGI_tinGut_0.0 |
| Accipitriformes | Bald eagle | | *Haliaeetus leucocephalus* | 88X | 10K/670K | PRJNA237821 | BGI_halLeu_0.0 |
| Struthioniformes | African ostrich | | *Struthio camelus* | 85X | 29K/3.5M | PRJNA212875 | BGI_strCam_0.0 |
| Passeriformes | American crow | | *Corvus brachyrhynchos* | 80X | 24K/6.9M | PRJNA212869 | BGI_corBra_0.0 |
| Pelecaniformes | Little egret | | *Egretta garzetta* | 74X | 24K/3.1M | PRJNA232959 | BGI_egrGar_0.0 |
| Columbiformes | Domestic pigeon | | *Columba livia* | 63X | 22K/3.2M | PRJNA167554 | BGI_colLiv_0.0 |
| Sphenisciformes | Emperor penguin | | *Aptenodytes forsteri* | 60X | 30K/5.1M | PRJNA235982 | BGI_aptFor_0.0 |
| Sphenisciformes | Adeliae penguin | | *Pygoscelis adeliae* | 60X | 19K/5.0M | PRJNA235983 | BGI_pygAde_0.0 |
| Anseriformes | Peking duck | | *Anas platyrhynchos* | 50X | 26K/1.2M | PRJNA46621 | BGI_duck_1.0 |
| **Low-coverage genomes** | | | | | | | |
| Passeriformes | | Rifleman | *Acanthisitta chloris* | 29X | 18K/64K | PRJNA212877 | BGI_acaChl_0.0 |
| Trogoniformes | | Bar-tailed trogon | *Apaloderma vittatum* | 28X | 19K/56K | PRJNA212878 | BGI_apaVit_0.0 |
| Gruiformes | | Grey crowned-crane | *Balearica regulorum* | 33X | 18K/51K | PRJNA212879 | BGI_balReg_0.0 |
| Bucerotiformes | | Rhinoceros hornbill | *Buceros rhinoceros* | 35X | 14K/51K | PRJNA212887 | BGI_bucRhi_0.0 |
| Caprimulgiformes | | Chuck-will’s-widow | *Caprimulgus carolinensis* | 30X | 17K/45K | PRJNA212888 | BGI_antCar_0.0 |
| Cariamiformes | | Red-legged seriema | *Cariama cristata* | 24X | 17K/54K | PRJNA212889 | BGI_carCri_0.0 |
| Accipitriformes | | Turkey vulture | *Cathartes aura* | 25X | 12K/35K | PRJNA212890 | BGI_catAur_0.0 |
| Gruiformes | | Macqueen’s bustard | *Chlamydotis macqueenii* | 27X | 18K/45K | PRJNA212891 | BGI_chlMac_0.0 |
| Coliiformes | | Speckled mousebird | *Colius striatus* | 27X | 18K/45K | PRJNA212892 | BGI_colStr_0.0 |
| Gruiformes | | Sunbittern | *Eurypyga helias* | 33X | 16K/46K | PRJNA212893 | BGI_eurHel_0.0 |
| Procellariiformes | | Northern fulmar | *Fulmarus glacialis* | 33X | 17K/46K | PRJNA212894 | BGI_fulGla_0.0 |
| Gaviiformes | | Red-throated loon | *Gavia stellata* | 33X | 16K/45K | PRJNA212895 | BGI_gavSte_0.0 |
| Accipitriformes | | White-tailed eagle | *Haliaeetus albicilla* | 26X | 20K/56K | PRJNA212896 | BGI_halAlb_0.0 |
| Leptosomiformes | | Cuckoo roller | *Leptosomus discolor* | 32X | 19K/61K | PRJNA212897 | BGI_lepDis_0.0 |
| Coraciiformes | | Carmine bee-eater | *Merops nubicus* | 37X | 20K/47K | PRJNA212898 | BGI_merNub_0.0 |
| Gruiformes | | Brown mesite | *Mesitornis unicolor* | 39X | 18K/46K | PRJNA212899 | BGI_mesUni_0.0 |
| Psittaciformes | | Kea | *Nestor notabilis* | 32X | 16K/37K | PRJNA212900 | BGI_nesNot_0.0 |
| Pelecaniformes | | Dalmatian pelican | *Pelecanus crispus* | 34X | 18K/43K | PRJNA212901 | BGI_pelCri_0.0 |
| Pelecaniformes | | White-tailed tropicbird | *Phaethon lepturus* | 39X | 18K/47K | PRJNA212902 | BGI_phaLep_0.0 |
| Pelecaniformes | | Great cormorant | *Phalacrocorax carbo* | 24X | 15K/48K | PRJNA212903 | BGI_phaCar_0.0 |
| Phoenicopteriformes | | American flamingo | *Phoenicopterus ruber* | 33X | 16K/37K | PRJNA212904 | BGI_phoRub_0.0 |
| Podicipediformes | | Great crested grebe | *Podiceps cristatus* | 30X | 13K/30K | PRJNA212905 | BGI_podCri_0.0 |
| Ciconiiformes | | Yellow-throated sandgrouse | *Pterocles gutturalis* | 25X | 17K/49K | PRJNA212906 | BGI_pteGut_0.0 |
| Musophagiformes | | Red-crested turaco | *Tauraco erythrolophus* | 30X | 18K/55K | PRJNA212908 | BGI_tauEry_0.0 |
| Strigiformes | | Barn owl | *Tyto alba* | 27X | 13K/51K | PRJNA212909 | BGI_tytAlb_0.0 |

^*^ Data source was derived from “Comparative genomics reveals insights into avian genome evolution and adaptation,” by Zhang et al., 2014, Science 346: 1311-1320.
