# Supplementary material for: Evolution of digestive enzymes and dietary diversification in birds
Source: PeerJ. 2019 Apr 25;7:e6840. doi: 10.7717/peerj.6840 (PMC6487185; doi:10.7717/peerj.6840)
Supplement: Table S3 [file peerj-07-6840-s011.docx]

| **Common name** | **Species name** | **Amylases** | **Carbohydrases except amylases** | **Lipases and proteases** | **Chitinase and lysozymes** |
| --- | --- | --- | --- | --- | --- |
| Turkey vulture | *Cathartes aura* |  |  | ✓ |  |
| White-tailed eagle | *Haliaeetus albicilla* |  |  | ✓ |  |
| Bald eagle | *Haliaeetus leucocephalus* |  |  | ✓ |  |
| Peking duck | *Anas platyrhynchos* | ✓ |  |  | ✓ |
| Anna’s hummingbird | *Calypte anna* |  | ✓ |  |  |
| Chimney swift | *Chaetura pelagica* |  |  |  | ✓ |
| Rhinoceros hornbill | *Buceros rhinoceros* |  | ✓ |  |  |
| Chuck-will’s-widow | *Caprimulgus carolinensis* |  |  |  | ✓ |
| Red-legged seriema | *Cariama cristata* |  |  |  | ✓ |
| Killdeer | *Charadrius vociferus* |  |  |  | ✓ |
| Crested ibis | *Nipponia nippon* |  |  | ✓ |  |
| Yellow-throated sandgrouse | *Pterocles gutturalis* | ✓ | ✓ |  |  |
| Speckled mousebird | *Colius striatus* |  | ✓ |  |  |
| Domestic pigeon | *Columba livia* | ✓ | ✓ |  |  |
| Carmine bee-eater | *Merops nubicus* |  |  |  | ✓ |
| Common cuckoo | *Cuculus canorus* |  |  |  | ✓ |
| Peregrine falcon | *Falco peregrinus* |  |  | ✓ |  |
| Chicken | *Gallus gallus* | ✓ | ✓ |  |  |
| Turkey | *Meleagris gallopavo* | ✓ | ✓ |  |  |
| Red-throated loon | *Gavia stellata* |  |  | ✓ |  |
| Grey crowned-crane | *Balearica regulorum* | ✓ |  |  | ✓ |
| Macqueen’s bustard | *Chlamydotis macqueenii* | ✓ | ✓ |  |  |
| Sunbittern | *Eurypyga helias* |  |  | ✓ | ✓ |
| Brown mesite | *Mesitornis unicolor* |  |  |  | ✓ |
| Cuckoo roller | *Leptosomus discolor* |  |  |  | ✓ |
| Red-crested turaco | *Tauraco erythrolophus* | ✓ | ✓ |  |  |
| Hoatzin | *Opisthocomus hoazin* |  |  |  |  |
| Rifleman | *Acanthisitta chloris* |  |  |  | ✓ |
| American crow | *Corvus brachyrhynchos* |  | ✓ | ✓ |  |
| Medium ground-finch | *Geospiza fortis* | ✓ | ✓ |  | ✓ |
| Golden-collared manakin | *Manacus vitellinus* |  | ✓ |  |  |
| Zebra finch | *Taeniopygia guttata* | ✓ | ✓ |  |  |
| Little egret | *Egretta garzetta* |  |  | ✓ | ✓ |
| Dalmatian pelican | *Pelecanus crispus* |  |  | ✓ |  |
| White-tailed tropicbird | *Phaethon lepturus* |  |  | ✓ |  |
| Great cormorant | *Phalacrocorax carbo* |  |  | ✓ |  |
| American flamingo | *Phoenicopterus ruber* | ✓ |  |  | ✓ |
| Downy woodpecker | *Picoides pubescens* |  |  |  | ✓ |
| Great crested grebe | *Podiceps cristatus* |  |  | ✓ |  |
| Northern fulmar | *Fulmarus glacialis* |  |  | ✓ | ✓ |
| Budgerigar | *Melopsittacus undulatus* | ✓ | ✓ |  |  |
| Kea | *Nestor notabilis* |  | ✓ |  |  |
| Emperor penguin | *Aptenodytes forsteri* |  |  | ✓ |  |
| Adeliae penguin | *Pygoscelis adeliae* |  |  |  | ✓ |
| Barn owl | *Tyto alba* |  |  | ✓ |  |
| African ostrich | *Struthio camelus* | ✓ | ✓ |  |  |
| White-throated tinamou | *Tinamus guttatus* |  | ✓ |  |  |
| Bar-tailed trogon | *Apaloderma vittatum* |  |  |  | ✓ |

For selection tests on a specified digestive enzyme, species denoted with a check mark (✓) indicates a higher consumption of particular dietary items, while others without the check mark indicates a lower consumption. For amylases, species were divided into two groups with contrasting seed ingestion; for carbohydrases except amylases, species were divided into two groups with contrasting ingestion of seeds, fruits and nectar; for lipases and proteases, species were divided into two groups with contrasting meat ingestion; for chitinase and lysozymes, species were divided into two groups with contrasting insect ingestion.
