# Supplementary material for: Evolution of digestive enzymes and dietary diversification in birds
Source: PeerJ. 2019 Apr 25;7:e6840. doi: 10.7717/peerj.6840 (PMC6487185; doi:10.7717/peerj.6840)
Supplement: Table S4 [file peerj-07-6840-s012.docx]

| ***chit1*** | **Upstream gene (*mybph*)** | **Downstream gene (*btg2*)** |
| --- | --- | --- |
| *Acanthisitta chloris* | scaffold3197 (2426..8119) | scaffold25871 (709..1047) |
| *Anas platyrhynchos* | scaffold297 (1659987..1670017, complement) | scaffold1119 (31006..46289, complement) |
| *Apaloderma vittatum* | scaffold36561 (2..9068) | incomplete sequencing |
| *Aptenodytes forsteri* | Scaffold213 (274716..284291) | Scaffold142 (3913338..3913697, complement) |
| *Balearica regulorum* | scaffold38864 (184..8616) | incomplete sequencing |
| *Buceros rhinoceros* | scaffold30165 (25338..33872, complement) | scaffold12448 (988..1350) |
| *Calypte anna* | **scaffold166** (541937..545437) | **scaffold166** (1963791..1964626, complement) |
| *Caprimulgus carolinensis* | scaffold34187 (23559..33074, complement) | scaffold38863 (1547..1906) |
| *Cariama cristata* | scaffold17830 (30105..39203, complement) | scaffold28470 (932..1288) |
| *Cathartes aura* | scaffold2426 (13478..22851) | C13520597 (2828..3187, complement) |
| *Chaetura pelagica* | scaffold470 (221430..231653) | scaffold152 (689439..690333) |
| *Charadrius vociferus* | scaffold333 (213771..223645) | scaffold266 (2307086..2309560, complement) |
| *Chlamydotis macqueenii* | scaffold23431 (140..9590) | scaffold29789 (7212..7556, complement) |
| *Colius striatus* | scaffold15269 (2..8583) | scaffold28110 (947..1279) |
| *Columba livia* | scaffold176 (532339..540706) | scaffold454 (223335..227560) |
| *Corvus brachyrhynchos* | scaffold555 (378518..387748) | scaffold72 (1809163..1811126) |
| *Cuculus canorus* | scaffold284 (1773254..1782921, complement) | scaffold495 (592482..593988, complement) |
| *Egretta garzetta* | scaffold202 (215753..226529) | scaffold105 (1883480..1884925) |
| *Eurypyga helias* | scaffold21510 (183..9069) | scaffold46684 (7041..7400, complement) |
| *Falco peregrinus* | scaffold21.1 (1220978..1230421, complement) | scaffold409.1 (454504..455997) |
| *Fulmarus glacialis* | scaffold27868 (244..9163, complement) | scaffold27844 (6512..6871, complement) |
| *Gallus gallus* | **Chr26** (1011100..1020608, complement) | **Chr26** (5122814..5126864) |
| *Gavia stellata* | scaffold19626 (30889..38875, complement) | incomplete sequencing |
| *Geospiza fortis* | scaffold26 (315721..324456) | scaffold14 (34847..38282, complement) |
| *Haliaeetus albicilla* | scaffold9786 (25509..35095, complement) | incomplete sequencing |
| *Haliaeetus leucocephalus* | Scaffold475 (1311713..1321096, complement) | Scaffold482 (2242163..2242522) |
| *Leptosomus discolor* | scaffold15984 (30284..34005, complement) | scaffold39054 (7305..7664, complement) |
| *Manacus vitellinus* | scaffold753 (31411..34806) | scaffold761 (1205930..1207820) |
| *Meleagris gallopavo* | **Chr28** (122385..132122, complement) | **Chr28** (4849949..4855107) |
| *Melopsittacus undulatus* | scf900160276468 (466329..475186, complement) | scf900160276904 (1203865..1205111) |
| *Merops nubicus* | incomplete sequencing | incomplete sequencing |
| *Mesitornis unicolor* | scaffold5982 (44634..53858) | scaffold38918 (188..1525, complement) |
| *Nestor notabilis* | scaffold24040 (2228..9360) | scaffold24395 (764..1144) |
| *Nipponia nippon* | Scaffold243 (1365358..1374882, complement) | Scaffold415 (1540381..1542163, complement) |
| *Opisthocomus hoazin* | scaffold489 (545211..552092, complement) | scaffold714 (6049..6408, complement) |
| *Pelecanus crispus* | scaffold40218 (339..9901) | incomplete sequencing |
| *Phaethon lepturus* | scaffold44165 (278..9732) | scaffold25768 (7437..8882, complement) |
| *Phalacrocorax carbo* | scaffold15220 (2..9193) | scaffold27954 (942..1301) |
| *Phoenicopterus ruber* | incomplete sequencing | incomplete sequencing |
| *Picoides pubescens* | scaffold932 (679..9610) | scaffold458 (1143514..1145580) |
| *Podiceps cristatus* | scaffold30870 (30588..39847, complement) | scaffold49643 (844..1329) |
| *Pterocles gutturalis* | scaffold27015 (84..8879) | scaffold36263 (3..1345) |
| *Pygoscelis adeliae* | Scaffold472 (217204..226629) | Scaffold197 (1564661..1564981, complement) |
| *Struthio camelus* | scaffold162 (194943..199432) | scaffold656 (130377..131700, complement) |
| *Taeniopygia guttata* | 197863223 (454..4254) | 197838129 (785..1129, complement) |
| *Tauraco erythrolophus* | scaffold4395 (31947..40918, complement) | scaffold23241 (1004..1363) |
| *Tinamus guttatus* | scaffold1734 (76095..78683, complement) | scaffold3037 (39851..40198, complement) |
| *Tyto alba* | scaffold10418 (5..9754) | scaffold30542 (6655..7020, complement) |
| ***lipf*** | **Upstream gene (*rnls*)** | **Downstream gene (*lipm*)** |
| *Acanthisitta chloris* | scaffold6307 (592..33971) | scaffold24658 (25490..33853, complement) |
| *Anas platyrhynchos* | **scaffold517** (211932..287846) | **scaffold517** (177286..182634) |
| *Apaloderma vittatum* | incomplete sequencing | incomplete sequencing |
| *Aptenodytes forsteri* | **Scaffold320** (276053..354742) | **Scaffold320** (235788..240414) |
| *Balearica regulorum* | scaffold7444 (20340..44642, complement) | incomplete sequencing |
| *Buceros rhinoceros* | incomplete sequencing | scaffold4675 (5393..14695, complement) |
| *Calypte anna* | **scaffold258** (4416297..4483221) | **scaffold258** (2670042..2679485, complement) |
| *Caprimulgus carolinensis* | scaffold12651 (13599..36260) | scaffold7459 (24981..34685, complement) |
| *Cariama cristata* | scaffold14494 (1671..19726, complement) | scaffold26100 (6478..13415) |
| *Cathartes aura* | scaffold123 (1295..71437, complement) | incomplete sequencing |
| *Chaetura pelagica* | **scaffold251** (930487..1005320, complement) | **scaffold251** (1033087..1041148) |
| *Charadrius vociferus* | **scaffold390** (1605401..1680777, complement) | **scaffold390** (1751700..1758790) |
| *Chlamydotis macqueenii* | incomplete sequencing | incomplete sequencing |
| *Colius striatus* | scaffold15346 (42747..55055, complement) | scaffold14840 (6195..14643, complement) |
| *Columba livia* | **scaffold171** (2449476..2530799, complement) | **scaffold171** (2549598..2556300) |
| *Corvus brachyrhynchos* | **scaffold334** (2669228..2721748, complement) | **scaffold334** (2739765..2743587, complement) |
| *Cuculus canorus* | **scaffold405** (414005..492900, complement) | **scaffold405** (523944..529990) |
| *Egretta garzetta* | **scaffold234** (209080..271006) | **scaffold234** (166466..174005, complement) |
| *Eurypyga helias* | scaffold1518 (12203..29592, complement) | scaffold1257 (142208..151230) |
| *Falco peregrinus* | **scaffold140.2** (973328..1048113) | **scaffold140.2** (942609..947716) |
| *Fulmarus glacialis* | incomplete sequencing | incomplete sequencing |
| *Gallus gallus* | **Chr6** (10113401..10178635) | **Chr6** (10101961..10109611) |
| *Gavia stellata* | scaffold44657 (20488..22006, complement) | scaffold4052 (113429..121068) |
| *Geospiza fortis* | **scaffold89** (209916..262483, complement) | **scaffold89** (313750..320477) |
| *Haliaeetus albicilla* | scaffold691 (6025..27593) | scaffold5616 (877..13186) |
| *Haliaeetus leucocephalus* | **Scaffold1249** (190967..271764, complement) | **Scaffold1249** (283885..288528, complement) |
| *Leptosomus discolor* | scaffold3504 (25265..2916, complement) | scaffold9712 (114847..122439) |
| *Manacus vitellinus* | **scaffold437** (297811..360137, complement) | **scaffold437** (411101..416955) |
| *Meleagris gallopavo* | **Chr8** (4633741..4647702, complement) | **Chr8** (4697465..4703268) |
| *Melopsittacus undulatus* | scf900160277070 (11855426..11927694, complement) | scf900160276939 (364465..374437) |
| *Merops nubicus* | scaffold4210 (829..1860) | scaffold10304 (43549..49580) |
| *Mesitornis unicolor* | scaffold8848 (10174..28750, complement) | scaffold13699 (8245..13855) |
| *Nestor notabilis* | scaffold24693 (2795..19713) | scaffold4559 (2902..10735) |
| *Nipponia nippon* | **Scaffold55** (11339520..11417278, complement) | **Scaffold55** (11436681..11441597, complement) |
| *Opisthocomus hoazin* | **scaffold64** (278084..355779) | **scaffold64** (266887..271188) |
| *Pelecanus crispus* | scaffold3327 (69433..87613, complement) | scaffold19113 (3316..9238, complement) |
| *Phaethon lepturus* | scaffold16002 (4118..23018, complement) | scaffold45527 (10784..15888) |
| *Phalacrocorax carbo* | incomplete sequencing | incomplete sequencing |
| *Phoenicopterus ruber* | incomplete sequencing | scaffold21386 (8261..16373, complement) |
| *Picoides pubescens* | scaffold2108 (3216..37461) | scaffold283 (6520673..6530125) |
| *Podiceps cristatus* | scaffold18131 (15561..35368, complement) | scaffold29451 (44637..49056) |
| *Pterocles gutturalis* | scaffold23308 (3809..22567, complement) | incomplete sequencing |
| *Pygoscelis adeliae* | **Scaffold139** (1859512..1941124, complement) | **Scaffold139** (2001412..2008490) |
| *Struthio camelus* | scaffold379 (20021..47408) | scaffold58 (94816..102070) |
| *Taeniopygia guttata* | 197858533 (1944..51918, complement) | 197858518 (2965..9704) |
| *Tauraco erythrolophus* | scaffold10796 (2..2386) | scaffold21172 (7567..14968, complement) |
| *Tinamus guttatus* | scaffold4836 (207998..265975, complement) | scaffold8773 (59814..66473, complement) |
| *Tyto alba* | scaffold8132 (5288..6452) | incomplete sequencing |
| **salivary *amy*** | **Upstream gene (*rnpc3*)** | **Downstream gene (*ntng1*)** |
| *Acanthisitta chloris* | scaffold44139 (6202..14735, complement) | scaffold36106 (14985..71376) |
| *Anas platyrhynchos* | scaffold2003 (692355..703008) | scaffold1979 (933117..1085764) |
| *Apaloderma vittatum* | scaffold7479 (163..10243) | scaffold39696 (25228..26646, complement) |
| *Aptenodytes forsteri* | **Scaffold181** (3597837..3607520, complement) | **Scaffold181** (2388780..2551862, complement) |
| *Balearica regulorum* | scaffold43990 (3832..17887) | scaffold381 (6136..157754) |
| *Buceros rhinoceros* | incomplete sequencing | scaffold30966 (4867..66662, complement) |
| *Calypte anna* | scaffold285 (378532..390620, complement) | scaffold79 (4114718..4265083, complement) |
| *Caprimulgus carolinensis* | scaffold10241 (14489..24826, complement) | scaffold5093 (6067..153924) |
| *Cariama cristata* | scaffold43683 (6869..17709, complement) | scaffold1107 (19181..81515, complement) |
| *Cathartes aura* | incomplete sequencing | scaffold38936 (1457..92969, complement) |
| *Chaetura pelagica* | **scaffold69** (11736534..11749276) | **scaffold69** (12594363..12744520) |
| *Charadrius vociferus* | **scaffold163** (18661142..18671569) | **scaffold163** (19710263..19862530) |
| *Chlamydotis macqueenii* | scaffold21554 (892..11667) | incomplete sequencing |
| *Colius striatus* | scaffold5105 (2773..13617, complement) | scaffold288 (139916..280438, complement) |
| *Columba livia* | **scaffold7** (5139022..5151050) | **scaffold7** (6037289..6184478) |
| *Corvus brachyrhynchos* | **scaffold101** (6524236..6533976, complement) | **scaffold101** (5514815..5636117, complement) |
| *Cuculus canorus* | **scaffold187** (1072704..1083335, complement) | **scaffold187** (11319..72677, complement) |
| *Egretta garzetta* | scaffold149 (3483863..3494906) | scaffold655 (968143..1100727) |
| *Eurypyga helias* | scaffold11419 (1041..10044) | scaffold5560 (5723..92641) |
| *Falco peregrinus* | **scaffold288.1** (3772168..3782630) | **scaffold288.1** (4795863..4946582) |
| *Fulmarus glacialis* | scaffold38923 (985..12425) | scaffold14826 (6081..130300) |
| *Gallus gallus* | **Chr8** (11468851..11483655, complement) | **Chr8** (1215502..1361252) |
| *Gavia stellata* | incomplete sequencing | scaffold38989 (13419..75629, complement) |
| *Geospiza fortis* | scaffold403 (6003776..6011530) | scaffold229 (530804..671064) |
| *Haliaeetus albicilla* | scaffold48520 (6363..16617, complement) | scaffold10295 (13479..75310, complement) |
| *Haliaeetus leucocephalus* | incomplete sequencing | Scaffold611 (317572..478517, complement) |
| *Leptosomus discolor* | scaffold7716 (1084..11857) | scaffold19195 (30850..81576) |
| *Manacus vitellinus* | **scaffold144** (3372484..3514847) | **scaffold144** (2489301..2498350) |
| *Meleagris gallopavo* | **Chr10** (10470347..10480384, complement) | **Chr10** (12770550..12892206) |
| *Melopsittacus undulatus* | scf900160276522 (1302925..1315784, complement) | scf900160277042 (6370209..6534960, complement) |
| *Merops nubicus* | scaffold21301 (922..10989) | scaffold26549 (799..30460) |
| *Mesitornis unicolor* | incomplete sequencing | scaffold4248 (6041..128280) |
| *Nestor notabilis* | scaffold7878 (13821..25621, complement) | scaffold25095 (20048..80270, complement) |
| *Nipponia nippon* | Scaffold2028 (11829..22831, complement) | Scaffold118 (452882..604394, complement) |
| *Opisthocomus hoazin* | scaffold44 (761418..772120, complement) | scaffold542 (395512..564197, complement) |
| *Pelecanus crispus* | scaffold16740 (2035..12337) | scaffold19153 (20436..25928) |
| *Phaethon lepturus* | scaffold38375 (1138..11817) | scaffold1640 (7808..99765, complement) |
| *Phalacrocorax carbo* | incomplete sequencing | scaffold14622 (22838..29184, complement) |
| *Phoenicopterus ruber* | incomplete sequencing | incomplete sequencing |
| *Picoides pubescens* | **scaffold171** (226045..226103) | **scaffold171** (1155002..1309103) |
| *Podiceps cristatus* | incomplete sequencing | scaffold3181 (5766..68278) |
| *Pterocles gutturalis* | incomplete sequencing | scaffold39621 (9242..73861, complement) |
| *Pygoscelis adeliae* | Scaffold10 (672806..682564, complement) | Scaffold265 (373856..530455) |
| *Struthio camelus* | scaffold540 (379702..389416) | scaffold252 (5828023..5955728, complement) |
| *Taeniopygia guttata* | incomplete sequencing | 197880498 (3696..61782) |
| *Tauraco erythrolophus* | scaffold6311 (7865..18696, complement) | scaffold24237 (9703..101619, complement) |
| *Tinamus guttatus* | scaffold274 (90070..90369) | scaffold1980 (59561..181118) |
| *Tyto alba* | incomplete sequencing | scaffold54633 (17358..19314, complement) |
