# Supplementary material for: Evolution of digestive enzymes and dietary diversification in birds
Source: PeerJ. 2019 Apr 25;7:e6840. doi: 10.7717/peerj.6840 (PMC6487185; doi:10.7717/peerj.6840)
Supplement: Table S5 [file peerj-07-6840-s013.docx]

| **Category** | **Gene** | **Sites** | **Site models (M8)^a^** | **Datamonkey** | | | **TreeSAAP properties^c^** |
| --- | --- | --- | --- | --- | --- | --- | --- |
|  |  |  |  | **FEL (*P* < 0.2)^b^** | **FUBAR (*PP* > 0.8)^b^** | **REL (BF > 50)^b^** | **Radical changes in AA properties** |
| Carbohydrases | hepatic *amy* | 3 |  | 0.05 |  | 436 | *pK'* |
|  |  | 64 |  | 0.05 |  | 222 | *pK'* |
|  |  | 66 |  | 0.07 |  | 162 | *pK'* |
|  |  | 70 | 0.97 | 0.11 | 0.93 |  | *µ Br El F Ns RF Ra pHi* |
|  |  | 148 |  | 0.17 |  | 91 | *Bl F Pβ P Ra* |
|  |  | 162 |  | 0.18 |  | 64 | *αc* |
|  |  | 174 |  | 0.10 |  | 224 | *αc* |
|  |  | 281 |  | 0.16 |  | 74 | *pK'* |
|  |  | 367 |  | 0.03 | 0.93 |  | *αm Br El F RF pHi* |
|  |  | 369 |  | 0.08 |  | 113 | *pK'* |
|  |  | 477 | 0.99 | 0.02 | 0.99 |  | *El Esm F Ht Ns RF Ra pHi* |
|  |  | 481 |  | 0.06 | 0.92 |  | *Bl Br El F Hp Ns Pβ P Pc Pr RF Ra p pHi* |
|  |  | 504 |  | 0.06 |  | 174 | *pK'* |
|  |  | 508 |  | 0.16 |  | 77 | *Pβ pK'* |
|  | pancreatic *amy* | 70 |  | 0.17 |  | 201 | *αc Br F Ht P RF p* |
|  |  | 84 |  | 0.03 |  | 117 | *pK'* |
|  |  | 92 |  | 0.19 |  | 91 | *αc* |
|  |  | 239 |  | 0.03 |  | 281 | *Pc* |
|  |  | 362 |  | 0.10 | 0.91 | 124 | *RF* |
|  |  | 367 | 0.96 | 0.01 | 0.99 | 280 | *µ Bl Ca Esm F Hnc Mv Mw Pβ P Pc Ra V0 pHi* |
|  |  | 454 |  | 0.13 |  | 209 | *Br F p pK'* |
|  |  | 455 | 0.99 | 0.00 | 1.00 | 583 | *αc Pβ Pr RF p* |
|  |  | 504 |  | 0.02 | 0.90 | 325 | *pK'* |
| Lipases | *cyp7a1* | 3 | 0.98 | 0.17 | 0.94 | 8616 | *F Hp P RF Ra pK'* |
|  |  | 10 | 0.95 |  |  | 552 | *Pα* |
|  |  | 22 |  | 0.08 |  | 204 | *Br F RF h p pHi pK'* |
|  |  | 30 | 0.96 | 0.01 | 0.99 | 40071 | *αm Pc pHi* |
|  |  | 128 |  | 0.12 |  | 62 | *pHi* |
|  |  | 162 |  | 0.14 |  | 89 | *αc Br F Pα P Pc RF Ra h p pHi* |
|  |  | 318 |  | 0.01 | 0.94 | 726 | *Et Hnc pHi* |
|  |  | 321 |  | 0.14 |  | 146 | *Pα* |
|  |  | 327 | 0.92 | 0.03 | 0.99 | 60492 | *F Hp P RF Ra pK'* |
|  |  | 335 |  | 0.06 |  | 264 | *pK'* |
|  |  | 337 |  | 0.14 |  | 121 | *αc F Pα P Pc RF Ra* |
|  |  | 358 |  | 0.06 |  | 265 | *pK'* |
|  |  | 392 | 0.97 |  |  | 119 | *Et pHi* |
|  |  | 424 |  | 0.18 |  | 58 | *Pr* |
|  |  | 468 |  | 0.00 | 0.97 | 1382 | *Pα* |
|  |  | 501 | 0.94 |  |  | 559 | *Pα* |
|  | *pnlip* | 14 |  | 0.01 | 0.96 | 301 | *Br Ht Ns pK'* |
|  |  | 18 |  | 0.10 | 0.97 | 233 | *pHi* |
|  |  | 43 |  | 0.15 | 0.95 | 383 | *F Hnc Ht RF V0* |
|  |  | 48 |  | 0.12 | 0.96 | 377 | *αc K0 p pHi* |
|  |  | 54 |  | 0.08 | 0.95 | 1112 | *Br El Ht K0 Ns P Pc Ra pK'* |
|  |  | 57 |  | 0.01 | 0.96 | 147 | *Br P RF Ra h p pK'* |
|  |  | 65 |  | 0.12 | 0.96 | 604 | *αc K0 Ns p* |
|  |  | 66 | 1.00 | 0.00 | 1.00 | 2e+05 | *αc Bl Br El Et F Hp Ht Ns Pβ P Pc Pr RF Ra h p* |
|  |  | 73 |  | 0.01 | 0.95 | 99 | *Ns Pβ pK'* |
|  |  | 75 | 1.00 | 0.02 | 1.00 | 7543 | *Br Ht K0 Pβ Pc p* |
|  |  | 76 | 0.95 | 0.13 | 0.96 | 602 | *αc Bl Ca Esm F Pβ Pc V0* |
|  |  | 79 | 1.00 |  | 0.90 | 54 | *pHi* |
|  |  | 80 | 1.00 | 0.00 | 1.00 | 17644 | *αm El Esm F Ht K0 Mv Mw Ns Pβ Pr RF V0 p pHi* |
|  |  | 124 |  | 0.01 | 0.95 |  | *αc* |
|  |  | 140 |  | 0.01 | 0.95 | 300 | *pK'* |
|  |  | 155 |  | 0.14 |  | 166 | *Br El F RF p pK'* |
|  |  | 184 | 0.96 |  |  | 84 | *Br Et Hnc Ns h p pHi* |
|  |  | 205 |  | 0.00 | 0.96 | 359 | *Br El Ht Ns P Pc Ra pK'* |
|  |  | 224 |  | 0.01 | 0.94 | 72 | *Bl Br Ht Ns pK'* |
|  |  | 237 |  | 0.04 | 0.93 | 544 | *αc αm αn F Hp Ht K0 Ns P Pc Ra* |
|  |  | 250 | 0.91 | 0.02 | 0.99 | 4679 | *αc Br El F Hnc Hp Ns Pβ Pr RF Ra h p pHi* |
|  |  | 256 | 1.00 | 0.18 | 0.93 | 89 | *αc Pc* |
|  |  | 308 |  | 0.11 | 0.96 |  | *Br El Ns Pβ P Pc h p* |
|  |  | 310 |  | 0.06 | 0.92 | 231 | *pHi* |
|  |  | 312 | 1.00 | 0.08 | 0.93 | 141 | *Bl Ca Esm F Ns RF V0 pHi* |
|  |  | 318 | 1.00 | 0.06 | 0.96 | 140 | *αc αm Br Ca F Hnc K0 RF pHi* |
|  |  | 335 |  | 0.14 | 0.96 | 431 | *Br Ht Ns pK'* |
|  |  | 336 |  | 0.02 | 0.99 | 1310 | *pHi* |
|  |  | 340 | 1.00 | 0.05 | 0.93 | 125 | *Br Et F Ns h p* |
|  |  | 353 |  | 0.01 | 0.94 |  | *Ht K0 Pc* |
|  |  | 366 |  | 0.12 |  | 187 | *pHi* |
|  |  | 370 |  |  |  | 55 | *αc pHi* |
|  |  | 381 |  | 0.00 | 1.00 | 22261 | *αc Pc* |
|  |  | 390 | 0.99 | 0.04 | 0.98 | 1884 | *Ns Pβ Ra pK'* |
|  |  | 391 |  | 0.02 | 0.98 | 1800 | *pHi* |
|  |  | 408 |  | 0.17 |  | 65 | *Ns Pβ h p pK'* |
|  |  | 410 |  | 0.16 |  | 158 | *pK'* |
|  |  | 424 | 1.00 | 0.02 | 1.00 | 97 | *µ Br Ca El Esm Et F Hnc Ht Pβ RF Ra V0 p pHi pK'* |
|  |  | 425 |  | 0.17 |  | 67 | *pK'* |
|  |  | 454 |  |  | 0.94 | 134 | *αc pHi* |
| Proteases | *pgb1* | 14 |  | 0.03 | 0.91 | 280 | *αc Br El Et F Hp Ns Pr RF Ra c h p pK'* |
|  |  | 101 |  | 0.06 | 0.96 | 53 | *µ αc Bl Ca F Ht P Pc RF Ra V0* |
|  |  | 143 |  | 0.09 |  | 791 | *αc Ca Esm pHi* |
|  |  | 213 |  | 0.02 | 0.95 | 510 | *Br Et F Ns h p pHi* |
|  |  | 216 | 1.00 | 0.00 | 1.00 | 7179 | *F pHi* |
|  |  | 220 | 0.97 |  | 0.92 |  | *F Ns Pc pK'* |
|  |  | 234 | 0.93 | 0.00 | 0.99 | 31322 | *pK'* |
|  |  | 259 |  | 0.01 | 0.99 | 1061 | *αc αn Bl Br El Et F Hp K0 Ns P Pc Pr RF Ra h p* |
|  |  | 269 |  | 0.08 |  | 96 | *αc Et K0 Pc pHi* |
|  |  | 279 |  | 0.03 | 0.90 |  | *Ns pK'* |
|  |  | 284 |  | 0.07 |  | 105 | *αc αm Ca Esm RF pHi* |
|  |  | 296 |  | 0.02 | 0.94 | 342 | *αc Et pHi* |
|  | *pgb2* | 25 |  | 0.12 |  | 100 | *Bl F P Ra* |
|  |  | 31 |  | 0.00 | 0.99 | 91 | *µ Bl Br Et F Ns P Ra h pHi* |
|  |  | 37 |  | 0.10 |  | 120 | *Bl Br El F Hp Ns P RF Ra p* |
|  |  | 86 |  | 0.06 |  | 132 | *Pα* |
|  |  | 102 |  | 0.10 |  | 183 | *αc pHi* |
|  |  | 104 |  | 0.01 | 0.97 | 57 | *αc Ns Pα RF p pHi* |
|  |  | 108 | 0.99 | 0.00 | 0.99 |  | *F p* |
|  |  | 116 |  | 0.03 | 0.90 | 99 | *µ Mw V0* |
|  |  | 160 |  | 0.04 | 0.91 | 152 |  |
|  |  | 174 |  | 0.01 | 0.96 | 4534 | *Pα* |
|  |  | 179 |  | 0.08 |  | 129 |  |
|  |  | 212 | 0.91 | 0.20 |  |  | *αc* |
|  |  | 222 |  | 0.09 |  | 119 | *Br Ht Ns pK'* |
|  |  | 231 |  | 0.03 | 0.92 | 251 | *pK'* |
|  |  | 232 |  | 0.03 | 0.94 | 426 | *Ns* |
|  |  | 262 | 0.97 | 0.04 | 0.92 | 668 | *Pα* |
|  |  | 284 |  | 0.16 |  | 50 | *pK'* |
|  |  | 293 |  | 0.13 |  | 103 | *Pα* |
|  |  | 312 |  | 0.01 | 0.97 | 66 | *Br Ht Ns pK'* |
| Chitinase | *chia* | 220 |  | 0.16 | 0.91 | 54 |  |
|  |  | 397 |  | 0.05 | 0.90 |  | *Br Ns* |
|  |  | 402 | 1.00 |  |  | 3608 | *Br* |
|  |  | 439 |  | 0.05 | 0.98 |  | *αc Br F RF p* |
| Lysozymes | *lyz* | 21 |  | 0.20 |  | 211 | *Et F RF pHi* |
|  |  | 50 | 0.97 | 0.10 | 0.92 | 313 | *Br Et pHi* |
|  |  | 54 |  | 0.10 | 0.93 | 673 | *αc Et* |
|  |  | 58 | 0.92 |  |  | 60 | *µ Esm Et F Hnc Mv V0 pHi* |
|  |  | 86 |  | 0.15 |  | 206 | *Esm RF pHi* |
|  |  | 109 |  | 0.11 |  | 81 | *pHi* |
|  |  | 128 |  | 0.04 |  | 94 | *pHi* |
|  |  | 137 |  | 0.10 | 0.92 | 670 | *αc* |
|  | *lygA* | 6 |  | 0.01 | 0.96 |  | *Pα* |
|  |  | 11 | 0.99 |  |  | 85 | *Pα* |
|  |  | 21 |  | 0.03 | 0.93 | 865 | *αc F K0 Pα Ra pK'* |
|  |  | 24 | 0.94 |  |  | 538 | *pK'* |
|  |  | 27 |  | 0.05 | 0.92 |  | *Br Ht Pα* |
|  |  | 69 |  | 0.00 | 0.99 | 837 | *αc F pHi* |
|  |  | 83 | 1.00 | 0.15 |  | 1e+26 | *αc Bl F Hp Pα P Ra* |
|  |  | 99 |  | 0.04 | 0.91 |  | *Br Esm Et RF h p pHi* |
|  |  | 125 |  | 0.09 |  | 114 | *Ca Esm* |
|  |  | 131 | 1.00 | 0.00 | 1.00 | 3e+07 | *Br Esm Et RF h p pHi pK'* |
|  |  | 137 |  | 0.07 | 0.91 | 260 | *Pα pK'* |
|  | *lygB* | 8 | 0.94 | 0.11 | 0.98 | 208 | *F Pβ Pr RF Ra h p* |
|  |  | 33 | 0.99 | 0.01 | 1.00 | 1779 | *pHi* |
|  |  | 36 |  |  | 0.97 | 116 | *αc* |
|  |  | 67 |  |  | 0.90 | 86 | *El F Hp Pβ RF Ra p* |
|  |  | 81 | 1.00 | 0.00 | 1.00 | 12404 | *El Et F Hp Ns RF Ra h p* |
|  |  | 83 | 1.00 | 0.19 |  |  | *Et F Ns p pHi* |
|  |  | 103 | 0.95 | 0.00 | 1.00 | 9189 | *Pα pHi* |
|  |  | 110 | 1.00 |  |  | 242 | *pHi* |
|  |  | 128 | 1.00 | 0.06 | 0.97 | 93 | *Pα Pβ P Pc* |
|  |  | 129 | 1.00 | 0.07 | 0.96 | 86 | *Pα pHi* |
|  |  | 132 | 0.98 | 0.02 | 0.98 | 451 | *pHi* |
|  | *lygC* | 39 | 1.00 | 0.04 | 0.98 |  | *c pHi* |
|  |  | 43 |  | 0.05 | 0.92 |  | *F Ht Ns RF Ra h pK'* |
|  |  | 102 |  | 0.19 | 0.94 |  | *pHi* |
|  |  | 103 |  | 0.05 | 0.91 |  | *pHi* |

^a^ Codons identified as under positive selection, along with Bayesian (BEB) analysis PPs > 0.9 under M8 models.

^b^ Codons estimated by three methods in Datamonkey. Sites were filtered with a significance level < 0.2 for fixed-effect likelihood (FEL), posterior probability > 0.8 for fast unconstrained Bayesian approximation (FUBAR), or Bayes factor > 50 for random-effect likelihood (REL).

^c^ Codons with radical changes in amino acid properties under categories 6-8 estimated by TreeSAAP. Thirty-one physicochemical amino acid properties were as follows: α-helical tendencies (***Pα***); average number of surrounding residues (***Ns***); β-structure tendencies (***Pβ***); bulkiness (***Bl***); buriedness (***Br***); chromatographic index (***RF***); coil tendencies (***Pc***); composition (***c***); compressibility (***K0***); equilibrium Constant of ionization for COOH (***pK'***); helical contact area (***Ca***); hydropathy (***h***); isoelectric point (***pHi***); long-range nonbonded energy (***El***); mean r.m.s. fluctuation displacement (***F***); molecular volume (***Mv***); molecular weight (***Mw***); normal consensus hydrophobicity (***Hnc***); partial specific volume (***V0***); polar requirement (***Pr***); polarity (***p***); power to be the C-terminal of α-helix (***αc***); power to be the middle of α-helix (***αm***); power to be the N-terminal of α-helix (***αn***); refractive index (***µ***); short- and medium- range nonbonded energy (***Esm***); solvent accessible reduction ratio (***Ra***); surrounding hydrophobicity (***Hp***); thermodynamic transfer hydrophobicity (***Ht***); total nonbonded energy (***Et***); turn tendencies (***P***).
