# Supplementary material for: Evolution of digestive enzymes and dietary diversification in birds
Source: PeerJ. 2019 Apr 25;7:e6840. doi: 10.7717/peerj.6840 (PMC6487185; doi:10.7717/peerj.6840)
Supplement: Table S6 [file peerj-07-6840-s014.docx]

| **Category** | **Gene** | **null^a^** | |  | **alternative^b^** | | | | **2△(ln *L*)^g^** | ***P* value** | **Corrected *P* value^h^** |
| --- | --- | --- | --- | --- | --- | --- | --- | --- | --- | --- | --- |
|  |  | **ln *L1*^c^** | **np1^d^** |  | **ω_1_^e^** | **ω_2_^f^** | **ln *L2*^c^** | **np2^d^** |  |  |  |
| Carbohydrases | hepatic *amy* | -9118.17 | 50 |  | 0.2098 | 0.1759 | -9117.47 | 51 | 1.41 | 0.2357 | 0.4190 |
|  | pancreatic *amy* | -9999.77 | 59 |  | **0.2296** | **0.1526** | -9995.07 | 60 | 9.40 | 0.0022 | **0.0176*** |
|  | *agl* | -28487.46 | 137 |  | 0.0826 | 0.088 | -28487.17 | 138 | 0.57 | 0.4519 | 0.6014 |
|  | *g6pc* | -7911.09 | 77 |  | 0.0845 | 0.0734 | -7911.60 | 78 | 1.03 | 0.3096 | 0.4534 |
|  | *gaa i* | -11234.16 | 74 |  | 0.0844 | 0.0653 | -11232.30 | 75 | 3.72 | 0.0537 | 0.1432 |
|  | *gaa ii* | -8591.42 | 41 |  | 0.0748 | 0.0696 | -8591.30 | 42 | 0.24 | 0.6240 | 0.6656 |
|  | *gck* | -4126.18 | 56 |  | 0.0023 | 0.0093 | -4122.20 | 57 | 7.95 | 0.0048 | 0.0256 |
| Lipases | *cyp7a1* | -12579.65 | 128 |  | **0.2084** | **0.1401** | -12574.09 | 129 | 11.11 | 0.0009 | **0.0144*** |
|  | *pnlip* | -16050.45 | 95 |  | 0.2974 | 0.4015 | -16047.09 | 96 | 6.72 | 0.0095 | 0.0380 |
| Proteases | *pgb1* | -10817.67 | 77 |  | 0.2887 | 0.2662 | -10817.43 | 78 | 0.48 | 0.4886 | 0.6014 |
|  | *pgb2* | -9646.45 | 74 |  | 0.2766 | 0.2321 | -9645.54 | 75 | 1.81 | 0.1779 | 0.3558 |
| Chitinase | *chia* | -6051.47 | 32 |  | 0.3112 | 0.224 | -6049.76 | 33 | 3.43 | 0.0641 | 0.1465 |
| Lysozymes | *lygA* | -6233.54 | 140 |  | 0.3081 | 0.3027 | -6233.53 | 141 | 0.02 | 0.8992 | 0.8992 |
|  | *lygB* | -4642.73 | 92 |  | 0.443 | 0.443 | -4642.85 | 93 | 0.25 | 0.6165 | 0.6656 |
|  | *lygC* | -4489.50 | 95 |  | 0.3757 | 0.3116 | -4488.99 | 96 | 1.02 | 0.3117 | 0.4534 |
|  | *lyz* | -4208.93 | 71 |  | 0.3085 | 0.4937 | -4206.76 | 72 | 4.35 | 0.0371 | 0.1187 |

^a^ In null model, all extant branches had the same ω and each ancestral branch had its own ω.

^b^ In alternative model, all extant branches were grouped into two lineages according to dietary components. For amylases, species were divided into two groups with contrasting seed ingestion; for carbohydrases except the amylases, species were divided into two groups with contrasting ingestion of seeds, fruits and nectar; for lipases and proteases, species were divided into two groups with contrasting meat ingestion; for chitinase and lysozymes, species were divided into two groups with contrasting insect ingestion. Each lineage had a unique ω (ω_1_ and ω_2_), and each ancestral branch had its own ω.

^c^ The natural logarithm of likelihood value.

^d^ Number of parameters.

^e^ The ω_1_ estimated for lineage containing species with relative higher consumption of particular dietary items for corresponding digestive enzyme gene.

^f^ The ω_2_ estimated for lineage containing species with relative lower consumption of particular dietary items for corresponding digestive enzyme gene.

^g^ Twice the difference in ln *L* between two models compared.

^h^ *P* value corrected by FDR method ([Benjamini and Hochberg 1995](#_ENREF_3)) and denoted with an asterisk (*) when ω estimated from lineage with higher consumption in a particular food component was significantly larger than that from lineage with lower consumption. Adaptive evolved genes were denoted with bold and underlined ω_1_, ω_2_ and corrected *P* values.
