# Supplementary material for: Evolution of digestive enzymes and dietary diversification in birds
Source: PeerJ. 2019 Apr 25;7:e6840. doi: 10.7717/peerj.6840 (PMC6487185; doi:10.7717/peerj.6840)
Supplement: Supplemental Material S1 [file peerj-07-6840-s016.docx]

**Supplementary Material for**

**Evolution of digestive enzymes and dietary diversification in birds**

Yan-Hong Chen, and Huabin Zhao^*^

Department of Ecology, Hubei Key Laboratory of Cell Homeostasis, College of Life Sciences, Wuhan University, Wuhan 430072, China

^*^Corresponding Authors:

Huabin Zhao

Department of Ecology, Hubei Key Laboratory of Cell Homeostasis,

College of Life Sciences, Wuhan University

299 Bayi Road, Wuhan, Hubei 430072, China

Email address: [huabinzhao@whu.edu.cn](mailto:huabinzhao@whu.edu.cn)

**Dietary data**

For a given species, the numerical value of each category represents the proportional usage of a given diet (**Fig. 1 and Table S1**). Given that most birds are general omnivores and the proportion of a particular component of food often varies significantly among species, we classified birds into two groups that contain species with relatively higher and lower consumption for one or multiple particular food components. Specifically, aiming to test whether the evolution of genes encoding proteases or lipases linked with meat consumption, birds were classified into two groups with contrasting meat consumption. In a similar fashion, aiming to investigate the evolution of amylases, species were divided into two groups with contrasting seed consumption; to investigate the evolution of carbohydrases except the amylases, species were divided into two groups with contrasting consumption of seeds, fruits or nectar; to investigate the evolution of enzymes involved in digesting insects, species were classified into two groups with contrasting insect consumption.

**Identification of digestive enzyme genes**

First, the complete protein sequences of each digestive enzyme gene were obtained from human, mouse, rat, alligator, chicken, turkey and zebra finch, and were used as queries for performing tblastn searches against each of 48 avian genomic sequences with the cutoff of e-value at 1e-10. Second, redundant sequences that hit the same genome regions were removed. Third, the retained blast hits mapping on the same scaffold were extended in both 5’ and 3’ directions and the exon/intron boundaries were predicted by the program GeneWise ([Birney, Clamp & Durbin 2004](#_ENREF_2)). Fourth, coding sequences shorter than 50% of the full length were discarded. Sequences covering the full sequences of query data with a putative start and stop codon were regarded as intact genes; those with partial coverage against the coding sequences from queries, were regarded as partial genes, which were characterized by an open reading frame (ORF); those with an interrupted reading frame were considered as pseudogenes. Fifth, the newly obtained genes were used as queries to conduct tblastn searches against the genomes, with the aim to identify additional genes. All candidate genes were ultimately verified by TBLASN searches against the entire GenBank database with their best blast hits as the known genes of targets.

**Phylogenetic analysis**

For each type of digestive enzyme genes, protein sequences deduced from nucleotide sequences of all intact genes were first aligned with the program MUSCLE version 3.6 ([Edgar 2004](#_ENREF_4)), and the alignments were subjected to manual inspection in MEGA version 6 ([Tamura et al. 2013](#_ENREF_10)). Next we conducted codon alignment with PRANK version 130410 ([Loytynoja & Goldman 2008](#_ENREF_5)) and removed gaps from multiple sequence alignments using Gblocks version 0.91b ([Castresana 2000](#_ENREF_3)). Given that most partial *chia* genes cover the majority of intact sequences, partial *chia* genes span exon 4 to exon 10 (918 bp, adding to 64% of the total length), these partial genes were retained and combined with intact *chia* genes for phylogenetic reconstruction. Likewise, due to the difficulty to find out the complete sequences of one or multiple exons in other digestive enzyme genes, partial coding sequences spanning exon 2 to exon 33 of the *agl* (3,921 bp, adding to 85% of the total length), exon 3 to exon 19 of the *gaa* (1,800 bp, adding to 64% of the total length), and exon 2 to exon 8 of the *gck* (972 bp, adding to 70% of total length) were retained for subsequent analyses.

**Selective pressure analyses**

In these analyses, the species tree derived from Prum et al. (2015) was used as the input tree for those single-copy genes, and the gene tree reconstructed by the Bayesian approach was used as the input tree for multi-copy *chia* or *lyz* (**Fig. S1 and S2**). In addition, evolutionary analyses of multi-copiy *lyg* and *gaa* were separately performed based on each subclade, namely *lygA*, *lygB*, *lygC*, *gaa i* and *gaa ii*, with the species tree applied as the input tree for each subclade (**Fig. S3 and Fig. S4)**. Similarly, the pepsinogen *C* gene (*pgc*) was found to have two copies in most birds and was separately analyzed for each copy (we referred the nomenclature proposed by Castrol et al (2012) and renamed each copy as *pgb1* and *pgb2*), with the species tree as the input tree for each copy (**Fig. S5)**. Note that since all identified intact *ctrc* sequences were derived from species with no meat in their food composition (**Fig. 1 and Table S1**), we did not perform evolutionary analysis on this gene, which is required to classify species into two groups with relatively higher or lower meat consumption.

When the two site-specific models (M8 and M8a) were compared, all sites under positive selection were identified using the Bayes Empirical Bayes (BEB) method with a cutoff of posterior probabilities ≥ 0.9 ([Yang 2007](#_ENREF_13)), and the likelihood ratio tests (LRT) between the two site models were conducted at a threshold of *P*<0.05. Simultaneously, three improved likelihood methods in the Datamonkey web server, with both nonsynonymous and synonymous substitution rates under consideration ([Pond & Frost 2005](#_ENREF_7)), were used to further assess the signals of positive selection. Of the three methods, the fixed-effect likelihood (FEL) is a statistically rigorous method with both independent d_N_ and d_S_ inferred to each site in the context of codon substitution models ([Pond & Frost 2005](#_ENREF_7)); the random-effect likelihood (REL) is an improvement of the Nielsen-Yang approach, which utilizes flexible but not overly parameter-rich rate distributions and permits both d_S_ and d_N_ to vary across codons independently ([Pond & Muse 2005](#_ENREF_8)); the fast unconstrained Bayesian approximation (FUBAR), which can utilize several computational shortcuts to accelerate the detection of positive or purifying selection, and to relax the REL restrictions mentioned above, leads to improved robustness against model misspecification and allows analysis on large datasets ([Murrell et al. 2013](#_ENREF_6)). Sites with the significance level<0.2 for FEL, posterior probability>0.8 for FUBAR, or Bayes factor>50 for REL were chosen as candidates under positive selection. Finally, a complementary approach was adopted to identify changes in protein physicochemical properties using the TreeSAAP package ([Woolley et al. 2003](#_ENREF_12)), which performs goodness-of-fit and categorical statistical tests on 31 categories of structural and biochemical amino acid properties. For each gene, higher values (6-8) of each category of amino acid properties indicate more radical substitutions and more radical changes, which are signatures of adaptive evolution ([Sunagar et al. 2012](#_ENREF_9)).

The branch models performed by CODEML in the PAML package ([Yang 2007](#_ENREF_13)) were used to estimate ω values among branches. Due to inability to determine the dietary composition of ancestral lineages, the alternative model assigned an independent ω value to each ancestral branch. By contrast, the null model assumed that all branches possess a same ω value. The chi-square test was applied to examine the difference between the alternative and null models at a significant value of 0.05, using the false discovery rate (FDR) approach to adjust multiple testing ([Benjamini & Hochberg 1995](#_ENREF_1)).

The RELAX method (Wertheim et al. 2015) in the HyPhy package (Pond, Frost & Muse 2005) was used to test whether natural selection is intensified or relaxed along any of the branches in avian phylogeny. For each gene, we labeled branches with relatively lower consumption of particular food items as “test branches” and other branches were labeled as “reference branches”. The alternative model estimates the ω distribution for test branches or reference branches with the selection intensity parameter *k*, while the null model fixes the distribution of ω to be the same between the two categories of branches with *k* constrained to 1 ([Wertheim et al. 2015](#_ENREF_11)). If the *P* value is less than 0.05 (calculated by a LRT), it implies that natural selection on the test branches has been relaxed (*k* < 1) or intensified (*k* > 1) relative to the reference branches. Furthermore, the less constrained partitioned exploratory model, which permits the proportion of sites and ω from each category of branches to change regardless of the selection intensity parameter (*k*), was used to estimate the ω distribution for the two categories of branches. Intensified selection was inferred as ω away from neutrality (ω = 1), while relaxed selection was predicted as ω converging to neutrality (ω = 1).

**Functional divergence testing**

The coefficients of functional divergence (*θ_I_* and *θ_II_*) represent the levels of type-I and type-II divergence, respectively. Sites with a posterior probability (Q*_k_*)>0.95 were selected as functional divergence-related candidates. Chi-square distribution and normal distribution were applied to estimate the likelihood ratio of type-I analysis and the z-score of type-II analysis at a significant value of 0.05, respectively.

**References**

Benjamini Y, and Hochberg Y. 1995. Controlling the false discovery rate - a practical and powerful approach to multiple testing. *Journal of the Royal Statistical Society Series B-Methodological* 57:289-300.

Birney E, Clamp M, and Durbin R. 2004. GeneWise and genomewise. *Genome Research* 14:988-995.

Castresana J. 2000. Selection of conserved blocks from multiple alignments for their use in phylogenetic analysis. *Molecular Biology and Evolution* 17:540-552.

Edgar RC. 2004. MUSCLE: multiple sequence alignment with high accuracy and high throughput. *Nucleic Acids Research* 32:1792-1797.

Loytynoja A, and Goldman N. 2008. Phylogeny-aware gap placement prevents errors in sequence alignment and evolutionary analysis. *Science* 320:1632-1635.

Murrell B, Moola S, Mabona A, Weighill T, Sheward DJ, Pond SLK, and Scheffler K. 2013. FUBAR: A fast, unconstrained bayesian approximation for inferring selection. *Molecular Biology and Evolution* 30:1196-1205.

Pond SLK, and Frost SDW. 2005. Datamonkey: rapid detection of selective pressure on individual sites of codon alignments. *Bioinformatics* 21:2531-2533.

Pond SLK, and Muse SV. 2005. Site-to-site aariation of synonymous substitution rates. *Molecular Biology and Evolution* 22:2375-2385.

Sunagar K, Johnson WE, O'Brien SJ, Vasconcelos V, and Antunes A. 2012. Evolution of CRISPs associated with toxicoferan-reptilian venom and mammalian reproduction. *Molecular Biology and Evolution* 29:1807-1822.

Tamura K, Stecher G, Peterson D, Filipski A, and Kumar S. 2013. MEGA6: molecular evolutionary genetics analysis version 6.0. *Molecular Biology and Evolution* 30:2725-2729.

Wertheim JO, Murrell B, Smith MD, Pond SLK, and Scheffler K. 2015. RELAX: detecting relaxed selection in a phylogenetic framework. *Molecular Biology and Evolution* 32:820-832.

Woolley S, Johnson J, Smith MJ, Crandall KA, and McClellan DA. 2003. TreeSAAP: selection on amino acid properties using phylogenetic trees. *Bioinformatics* 19:671-672.

Yang ZH. 2007. PAML 4: Phylogenetic analysis by maximum likelihood. *Molecular Biology and Evolution* 24:1586-1591.
